# Glymphatic–meningeal lymphatic system imbalance: a peripheral-to-central inflammatory bridge in perioperative neurocognitive disorders

**DOI:** 10.3389/fimmu.2026.1828809

**Published:** 2026-04-21

**Authors:** Hongyou Wang, Xuedong Wang, Nan Liu, Heng Wang, Chunsheng Feng

**Affiliations:** 1 Department of Anesthesiology, The First Hospital of Jilin University, Changchun, China; 2 China–Japan Union Hospital, Jilin University, Changchun, China

**Keywords:** anesthesia, blood–brain barrier, glymphatic system, meningeal lymphatic vessels, neuroinflammation, perioperative neurocognitive disorders, surgery

## Abstract

Perioperative neurocognitive disorders (PNDs) are common postoperative complications, particularly in elderly patients. While surgical trauma is known to trigger systemic inflammation, the mechanisms linking peripheral immune activation to perioperative neurocognitive dysfunction remain not fully elucidated. The glymphatic–meningeal lymphatic system is crucial for maintaining homeostasis because it facilitates the exchange of cerebrospinal fluid and interstitial fluid, clears metabolic waste, and eliminates immune mediators. Recent studies have indicated that dysfunction of this clearance axis may contribute to the exacerbation of PNDs. This article explores how perioperative inflammation may influence the glymphatic–meningeal lymphatic system, thereby promoting neuroinflammation. We propose that the interplay between the inflammatory burden and the clearance capacity of the brain is a critical factor in the pathogenesis of PNDs. Through multimodal approaches—integrating advanced imaging techniques, high-dimensional immunogenomic profiling, biofluid biomarkers, and neurophysiological monitoring—we can more comprehensively characterize alterations in glymphatic–meningeal lymphatic function and their interactions with microcirculatory and immune dynamics. Furthermore, we discuss potential therapeutic strategies targeting the glymphatic–meningeal lymphatic system, which could offer clinical insights for the prevention and treatment of PNDs by targeting the underlying mechanisms.

## Graphical Abstract

**Figure:**
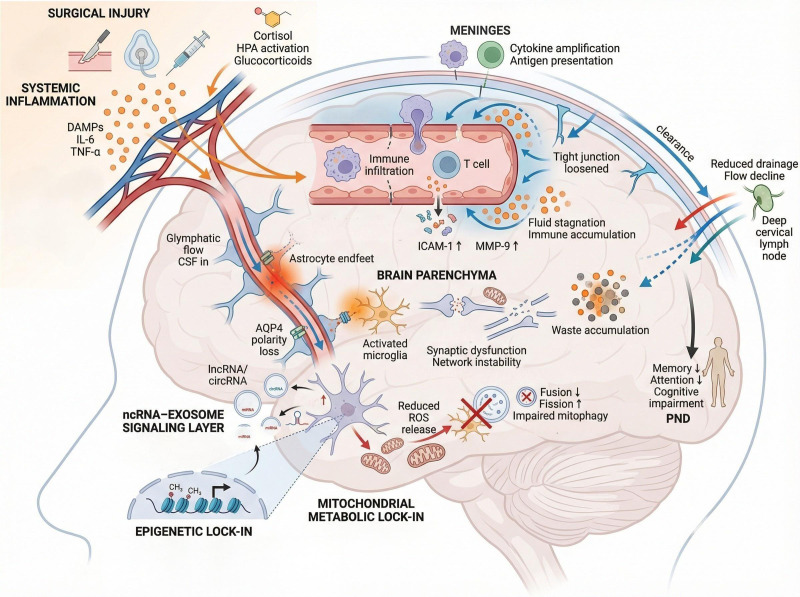
Mechanistic model of PNDs associated with glymphatic–mLV dysfunction. Surgical trauma and anesthesia induce systemic inflammation, compromise BBB integrity, and alter endothelial and perivascular function. These alterations disrupt CSF–ISF dynamics, compromise astrocytic aquaporin-4 (AQP4) polarization, and impair mLV drainage, collectively diminishing glymphatic–mLV-mediated clearance. The retention of inflammatory mediators and metabolic waste drives microglial activation, synaptic dysfunction, white matter vulnerability, and mitochondrial stress, resulting in the formation of a self-amplifying neuroinflammatory loop that culminates in PNDs.

## 1 Introduction

Perioperative neurocognitive disorders (PNDs) are common neurological complications associated with surgery or anesthesia. These complications manifest during various stages of the perioperative period and primarily affect attention, memory, executive function, and processing speed (1, 2). This framework encompasses preexisting neurocognitive disorders (NCDs), postoperative delirium (POD) occurring within 1–7 days following surgery or before discharge, delayed neurocognitive recovery (dNCR) lasting approximately 30 days postoperatively, and mild or major NCDs diagnosed between 30 days and 12 months after surgery (3). Epidemiological studies indicate that the risk of PNDs increases with age and surgical complexity, with prevalence rates ranging from 9% to 54% in individuals aged 65 years and older (2, 4, 5). PNDs are associated with prolonged hospital and ICU stays, an increased 30-day readmission risk, and both short-term and long-term cognitive decline. Furthermore, PNDs are correlated with decreased health-related quality of life, an increased risk of dementia, and increased long-term mortality rates (4, 6, 7).

Over the past three decades, the understanding of the pathogenesis of PNDs has evolved significantly. Initial studies attributed PNDs to the direct neurotoxic effects of anesthetics; however, this hypothesis fails to adequately explain the delayed onset, partial reversibility, and strong association with surgical trauma observed in the clinic (8, 9). Subsequent research has increasingly focused on the role of surgery-induced systemic inflammation and immune activation. Peripheral inflammatory mediators released after surgical trauma can disrupt blood–brain barrier (BBB) integrity, increase barrier permeability, and facilitate neuroinflammatory responses within the central nervous system (CNS), thereby contributing to perioperative cognitive impairment (10, 11). The dynamic regulation of BBB permeability has emerged as a critical immunological interface that links peripheral inflammatory signals to CNS responses (12). In recent years, with the discovery of the glymphatic system and meningeal lymphatic vessels (mLVs), the role of central clearance pathways in the transport of inflammatory mediators and immune-derived signals and the removal of metabolic waste has gradually attracted attention (13). In 2012, Iliff et al. introduced the concept of the glymphatic system, suggesting the existence of a cerebrospinal fluid–interstitial fluid (CSF–ISF) exchange pathway within the brain parenchyma (14). This framework supplemented the traditional arachnoid granulation–venous drainage model by incorporating an intracerebral component of CSF clearance (15). Later work validated the presence of mLVs in both animal models and human dural tissue, progressively elucidating their anatomical organization and role in the peripheral drainage of CSF and brain-derived solutes, as well as potentially immune-relevant antigens, toward deep cervical lymph nodes (dcLNs) (16, 17). Current evidence indicates that following CSF–ISF exchange, solutes within the brain parenchyma can enter the subarachnoid space and are primarily transported via mLVs, including parasagittal and skull base vessels, or through the olfactory nerve–cribriform plate pathway into the nasal-associated lymphatic network, ultimately converging on dcLNs (18, 19). Together, these pathways establish a structural and functional conduit between the CNS and the peripheral immune system.

In the context of increased perioperative inflammation, PND development may reflect the balance between peripheral inflammatory input to the brain and the efficiency of central clearance mechanisms. Increased BBB permeability augments inflammatory influx, whereas glymphatic–mLV dysfunction permits the persistence of neuroinflammatory mediators and immune-derived signals within the CNS. Therefore, this paper systematically reviews brain clearance reserve, analyzes the critical role of BBB dysfunction in enhancing inflammatory mediator influx, and explores the potential mechanisms of glymphatic–mLV system impairment in the development of PNDs, along with its multimodal assessment and intervention prospects.

## 2 The glymphatic–mLV system and central clearance reserve

The development of PNDs reflects the dynamic interplay between perioperative systemic perturbations and the intrinsic clearance capacity and neuroimmune homeostatic mechanisms of the brain. Within this context, the glymphatic–mLV system constitutes the structural basis of the central clearance reserve, which sets the functional threshold for tolerating perioperative systemic perturbations. Multiple intrinsic biological factors influence the efficiency of CSF–ISF exchange and mLV drainage, thereby shaping the ability of the brain to eliminate inflammatory mediators. When the inflammatory load surpasses the clearance capacity of the glymphatic–mLV system, homeostatic resolution may be impaired, resulting in sustained neuroimmune activation and increased vulnerability to PNDs.

### 2.1 Effects of genetic and developmental factors on the glymphatic–mLV system

Pediatric patients (particularly newborns and preterm infants) represent a special population exhibiting potentially increased perioperative neurocognitive vulnerability because the glymphatic–mLV system remains structurally and functionally immature during early life, with limited drainage pathways and clearance capacity being reported (20, 21). The diffusion tensor imaging along the perivascular space (DTI-ALPS) index, which is a surrogate imaging metric of perivascular water diffusion, is markedly lower in preterm infants than in term infants and increases with gestational and postnatal age, thus suggesting progressive functional maturation of the glymphatic–mLV system (22, 23). Animal studies have indicated that the distribution of astrocytic aquaporin-4 (AQP4), which is a key water channel that is enriched in perivascular astrocytic endfeet and essential for efficient cerebrospinal fluid–interstitial fluid exchange, does not achieve a fully polarized distribution during early development, thereby limiting the maturation and efficiency of the glymphatic–mLV system (20, 24–26). In this context, two hours of 3% sevoflurane anesthesia administered consecutively for three days induces cognitive impairment in juvenile mice, thus suggesting that limited clearance capacity during early development may amplify vulnerability to perioperative inflammatory stress (27). Clinically, children undergoing both cardiac and noncardiac surgery may exhibit postoperative cognitive dysfunction, with transient deficits in memory, attention, and visuomotor function being reported after general anesthesia; moreover, the incidence of early POCD ranges from low percentages to 15.6% (28–30). Together, these findings support the viewpoint that developmental immaturity of the glymphatic–mLV system may contribute to increased susceptibility to perioperative cognitive impairment in pediatric patients.

Genetic factors shape susceptibility to perioperative stress by modulating key structural and functional nodes of the glymphatic–mLV system of the brain. For example, the APOE ϵ4 allele is associated with reduced amyloid-beta (Aβ) clearance and impaired mLV function, whereas AQP4-related polymorphisms may alter perivascular water transport and glymphatic clearance efficiency (31, 32). The VEGF-C–VEGFR3 signaling axis is essential for mLV formation, and functional variants in *VEGF-C* or *VEGFR3* may restrict CSF drainage capacity (17, 33). In animal models, VEGF-C also attenuates microglia-mediated inflammatory responses by upregulating *Sik1* and inhibiting nuclear factor kappa B (NF-κB) signaling (34, 35). Genetic polymorphisms in *CRP* and *SELP* may attenuate perioperative systemic inflammation, thereby reducing the clearance burden on the glymphatic–mLV system (36, 37). In contrast, *PHLPP2* variants and reduced expression of brain-derived neurotrophic factor (BDNF) primarily compromise neuronal resilience to acute inflammation and metabolic waste accumulation in a time- and population-dependent manner (38, 39).

### 2.2 Age-related alterations in the glymphatic–mLV system associated with chronic comorbidities

Aging is among the most significant independent risk factors for PNDs (4, 40, 41). Although transient postoperative cognitive fluctuations may occur at any age, older adults are particularly vulnerable to persistent or pronounced cognitive deficits (4). Animal studies have demonstrated that age strongly modulates the cognitive effect of anesthesia; specifically, isoflurane induces hippocampal inflammation and spatial memory deficits in aged mice, whereas repeated exposure in adult rats results in minimal impairments (42, 43). Imaging studies have demonstrated an age-related decrease in glymphatic–mLV function, as evidenced by a marked reduction in the DTI-ALPS index (44). Dynamic MRI further confirmed that CSF influx into the brain interstitium decreases with age (45). Additional animal studies have revealed age-related structural alterations, including impaired mLV drainage, disrupted type IV collagen distribution in the basal meninges, reduced lymphatic valve density, and abnormal remodeling of lymphatic endothelial junctions from “zipper-like” to “button-like” configurations (46). Furthermore, decreased CSF production and degeneration of arachnoid villi collectively impair CSF–ISF convective exchange and mLV drainage efficiency (47, 48). The age-related disruption of astrocyte-dependent perivascular fluid transport is closely associated with impaired clearance of metabolic byproducts such as Aβ and alpha-synuclein, thereby aggravating ISF retention and creating a vicious cycle of clearance dysfunction (24, 25, 49, 50). Additionally, diminished activity of the VEGF-C–VEGFR3 signaling axis during aging accelerates the degeneration of mLVs and limits CSF drainage capacity (51).

Chronic metabolic and vascular comorbidities may further weaken the brain clearance reserve and thereby increase susceptibility to PNDs, particularly in older patients. Among these conditions, diabetes represents a clinically important high-risk subgroup. Experimental studies have demonstrated that diabetes impairs CSF clearance in the hippocampus and hypothalamus and is associated with cognitive decline (52). Consistent with this scenario, MRI studies in diabetic patients have demonstrated reduced CSF diffusion and perivascular flow (53, 54), thus supporting the presence of compromised glymphatic–mLV function. Clinically, elderly patients with diabetes exhibit a higher incidence of PND within 30 days after surgery than non-diabetic controls (59.2% vs. 36.8%) (55); moreover, these patients demonstrate an increased risk of delayed neurocognitive recovery after thoracic surgery (56) and may exhibit elevated postoperative delirium and persistent cognitive deficits for up to 9 months after elective noncardiac surgery (57). Mechanistically, persistent hyperglycemia may promote astrocytic dysfunction and disrupt molecular determinants of perivascular fluid exchange, thereby further aggravating clearance inefficiency and perioperative neurocognitive vulnerability (58).

Similarly, hypertension may increase PND susceptibility by impairing the hemodynamic support required for glymphatic–mLV function. Reduced arterial compliance and pulsatility weaken perivascular pumping, thereby restricting both CSF influx and efflux within the brain parenchyma (59, 60). Consistent with this clearance-related vulnerability, a history of hypertension has been identified as an independent risk factor for major POCD in elderly patients undergoing gastrointestinal tumor surgery (61).

In addition, some patients who are at high risk for PNDs may harbor preexisting neurodegenerative or neurovascular conditions, such as preclinical Alzheimer’s disease or cerebral microembolism, which can further compromise arterial pulsatility and glymphatic–mLV clearance (48, 62). These baseline deficits may remain subclinical under resting conditions; however, perioperative stressors can push the clearance system beyond its compensatory capacity, thereby converting latent vulnerability into overt postoperative cognitive impairment (41, 63).

### 2.3 Effects of sex and body temperature on the function of the glymphatic–mLV system

Sex differences in clearance mediated by the glymphatic–mLV system may manifest as variations in fluid transport efficiency and circadian rhythms. Although mouse models show limited sex differences in CSF inflow, imaging studies have indicated that females exhibit higher DTI-ALPS index than males do, suggesting a potentially greater perivascular fluid transport efficiency (64, 65).

Body temperature and metabolic status markedly influence central clearance efficiency during the perioperative period. General anesthesia can lower the brain temperature by ~3–4 °C, and hypothermia can transiently impair glymphatic–mLV clearance by reducing water diffusion and inhibiting lymphatic drainage (66, 67). Nevertheless, hypothermia also has clear neuroprotective effects, and its effect on perioperative neurological outcomes depends on the dynamic balance between clearance suppression and metabolic protection (68).

### 2.4 Microbial–gut–brain axis and central clearance function

Through interactions with the CNS, enteric nervous system (ENS), and enteroendocrine signaling, the gastrointestinal microenvironment regulates homeostasis and modulates susceptibility to central inflammation (69, 70). The microbiota–gut–brain axis forms a complex network involving neural, endocrine, metabolic, and immune pathways, with the glymphatic–mLV system potentially playing a key role in integrating these signals (70).

Intestinal inflammation and dysbiosis can directly impair CSF dynamics and glymphatic–mLV-mediated clearance (70). Specifically, the translocation of gut-derived pathogen-associated molecular patterns (PAMPs), such as Lipopolysaccharide (LPS), can prime proinflammatory T cells within gut-associated lymphoid tissue (71). Overactivation of the NLR family pyrin domain containing 3 (NLRP3) inflammasome induces neuroinflammation and disrupts astrocyte function, leading to a loss of AQP4 polarity and reduced CSF–ISF exchange, and may contribute to PNDs (72). Additionally, microbial metabolites can modulate astrocyte function and influence neuroinflammatory pathways (69). During gut dysbiosis, microbes and their metabolites stimulate the release of proinflammatory cytokines from intestinal epithelial cells and macrophages (70, 73). These systemic inflammatory signals, alongside recruited gut-primed immune cells, can enter the brain parenchyma via the glymphatic–mLV system, further amplifying central nervous system inflammation (74, 75).

**Figure 1:**
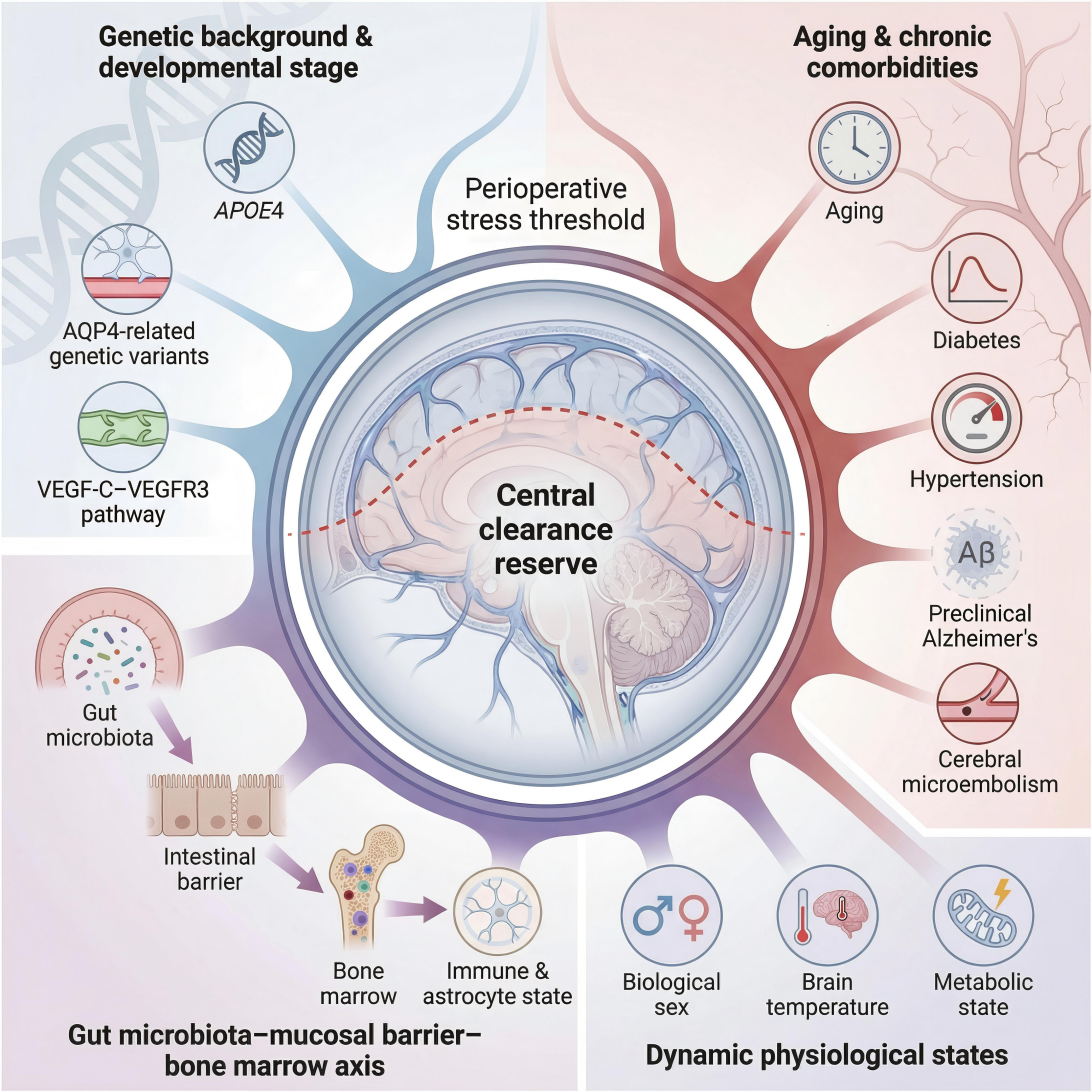
The central clearance reserve determines the perioperative stress threshold. The central clearance reserve is based on genetic and developmental factors, the persistent depletion caused by aging and chronic comorbidities, and the regulatory effects of the microbial–gut–brain axis and dynamic physiological states. The integration of these factors determines an individual’s tolerance threshold for the perioperative inflammatory load. When perioperative stress exceeds the threshold corresponding to an individual’s clearance reserve, it readily triggers a central inflammatory imbalance and cognitive impairment.

In summary, PNDs are strongly influenced by the functional reserve and regulatory capacity of the glymphatic–mLV system. When perioperative stressors further compromise an already limited reserve, inflammatory mediators and metabolic byproducts may accumulate in the central nervous system, thereby increasing the risk of PNDs (**Figure 1**).

## 3 The core role of BBB impairment in imbalances between perioperative inflammatory input and intracerebral clearance

The BBB is a key interface that regulates the entry of circulating factors into the brain, influencing the environment for the glymphatic–mLV system. Perioperative stressors disrupt BBB integrity, altering endothelial function, the perivascular space (PVS), and CSF–ISF exchange. These disturbances impair BBB–glymphatic–mLV coupling, ultimately contributing to glymphatic–mLV dysfunction.

### 3.1 Structural basis of the BBB as a glymphatic–mLV input

The BBB is a highly specialized interface within the central nervous system that is crucial for selective substance transport and the maintenance of homeostasis in the brain microenvironment (76). These functions depend not only on the cerebral capillary endothelium but also on coordinated regulation by the neurovascular unit (NVU) (77). Structurally, the BBB consists of a monolayer of brain microvascular endothelial cells with tight junctions whose integrity and vascular stability are supported by astrocytic endfeet, pericytes, and microglia. This architecture underlies and supports CNS homeostasis (12).

Under physiological conditions, the BBB not only restricts bloodborne molecules from entering the brain parenchyma but also regulates CSF–ISF exchange at the PVS, establishing an upstream structural interface for glymphatic–mLV function. Its selective permeability and vascular stability govern the exposure of inflammatory mediators in the PVS, thereby influencing the input load handled by this clearance pathway (78, 79). Perioperative models have revealed BBB alterations, including increased permeability and endothelial activation, creating conditions for peripheral inflammatory signals to act on the PVS and potentially altering the operational environment of the paracellular lymphatic route (80, 81).

### 3.2 Temporal stratification of BBB dysfunction during the perioperative period

BBB dysfunction plays a critical role in the onset and progression of PNDs (76, 82). On the basis of current evidence, perioperative alterations in BBB integrity can be classified into three sequential phases, with some overlap, each contributing differently to PND development.

The early phase (0–6 h) is characterized primarily by endothelial activation and increased functional permeability. Surgical stress induces the rapid release of proinflammatory mediators, including TNF-α, IL-1β, IL-6, and HMGB1, which activate BBB endothelial cells (83, 84). This activation upregulates adhesion molecules such as ICAM-1 and VCAM-1, leading to the disruption of tight junctions and increased permeability (70). HMGB1, as a damage-associated molecular pattern, binds to RAGE on endothelial cells, initiating the NF-κB pathway and further amplifying the inflammatory response, which exacerbates BBB permeability (63, 84, 85).

In the intermediate phase (6–24 h), the degradation of extracellular matrix components, such as collagen IV and laminin, mediated by MMP-9, destabilizes tight junctions, further enhancing BBB breakdown (86). Structural damage to the extracellular matrix, tight junctions, and pericellular structures amplifies neuroinflammation, leading to progressive breakdown of the barrier (12, 87–89).

The late phase (24–72 h) is characterized by sustained barrier opening, allowing peripheral CCR2^+^ monocytes to infiltrate brain tissue after transport through the compromised BBB, reshaping the local inflammatory microenvironment (5, 90, 91). Simultaneously, fibrinogen extravasates from the bloodstream and is converted to fibrin within the brain, where it can activate the TLR4/MyD88 signaling pathway, thereby amplifying neuroinflammation (92–94). The extravasation of fibrinogen and other plasma proteins increases interstitial hydraulic resistance, impairing glymphatic clearance and reducing the ability of the brain to clear neurotoxic substances, such as Aβ and tau proteins. This creates a neurotoxic environment that fosters chronic neuroinflammation and cognitive decline (95–97).

**Figure 2:**
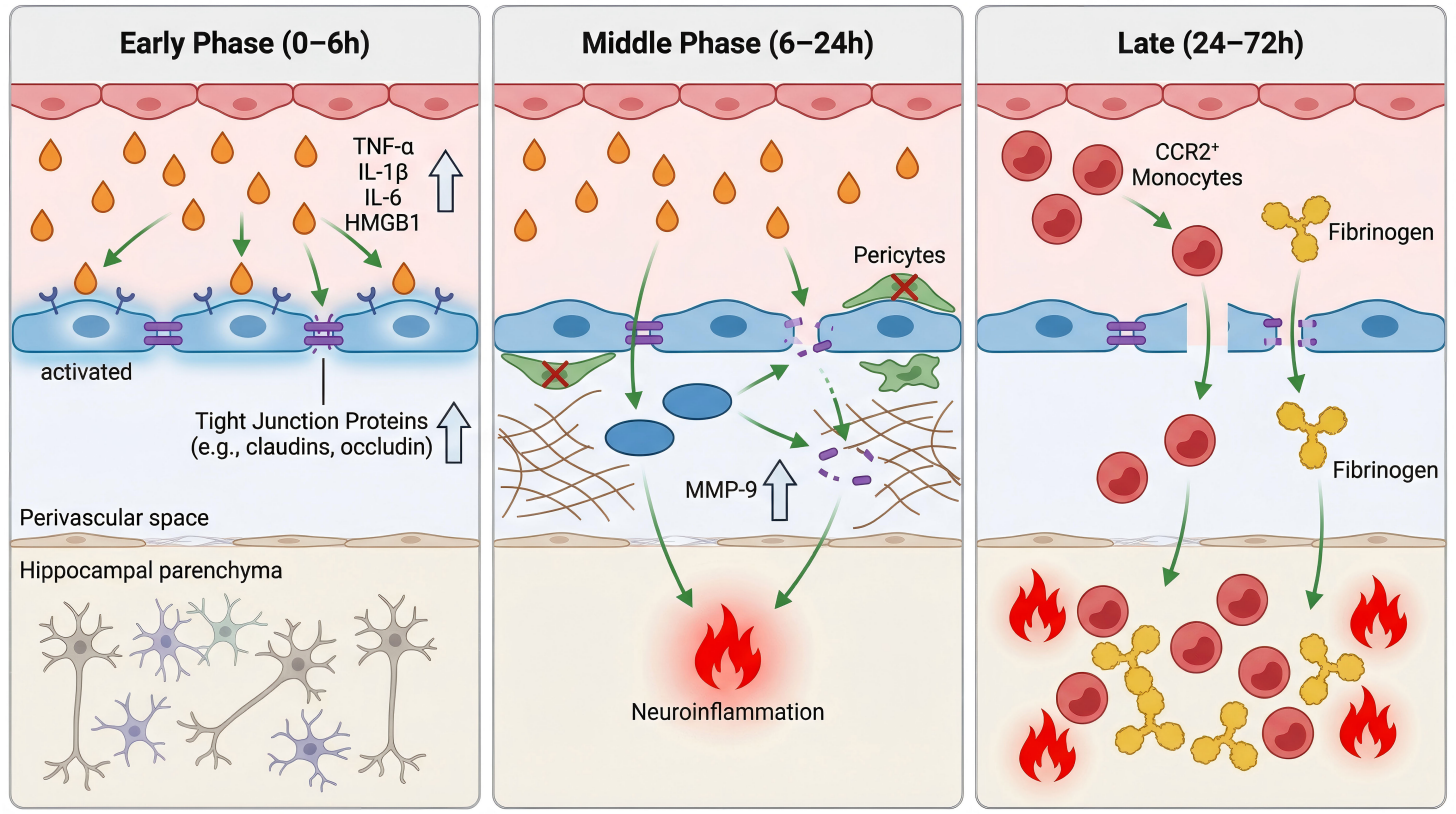
Time-dependent characteristics of perioperative BBB dysfunction. Perioperative BBB dysfunction progresses through early (0–6 h) endothelial activation, intermediate (6–24 h) structural remodeling, and late (24–72 h) sustained barrier opening with immune cell infiltration and macromolecule deposition.

This temporally stratified framework facilitates the integration of observations across different models and levels of evidence, providing a mechanistic coordinate system for deciphering the dynamic relationship between BBB dysfunction and neurocognitive outcomes (**Figure 2**).

### 3.3 Imbalanced coupling of the BBB–glymphatic–mLV system and PNDs

The perivascular space (PVS), which is continuous with the vascular basement membrane and the BBB, surrounds penetrating cerebral vessels and facilitates CSF–ISF exchange through the glymphatic–mLV system (22, 81). The BBB and glymphatic systems are highly interdependent: dysfunction in one leads to failure in the other (98–100). Perioperative stress increases BBB permeability, exposing the PVS to inflammatory mediators, which disrupt both BBB integrity and glymphatic clearance (93, 94, 101). Inflammatory cytokines such as IL-23, TNF-α, and IL-1β impair tight junction and endothelial permeability, exacerbating BBB dysfunction and disrupting astrocyte organization and AQP4 polarization (50, 102). This impairs fluid and waste clearance from the brain, creating a vicious cycle of inflammation and dysfunction. Persistent T-cell activation and monocyte differentiation into dendritic cells in the PVS promote further disruption of the BBB and increase local inflammation (103). Chemokines such as CCL2, CXCL10, and CXCL13 promote T-cell migration across the BBB, sustaining local inflammation and further disrupting CSF–ISF exchange, exacerbating glymphatic failure and neuroinflammation (104, 105). Vascular stability is crucial for BBB–glymphatic coupling (106). Inflammatory cytokines and impaired signaling of vascular support factors, such as PDGF-B and TGF-β, destabilize pericytes and weaken BBB integrity, compromising glymphatic efficiency (107, 108). This exacerbates both BBB dysfunction and glymphatic failure, accelerating the progression of PNDs (109).

**Figure 3:**
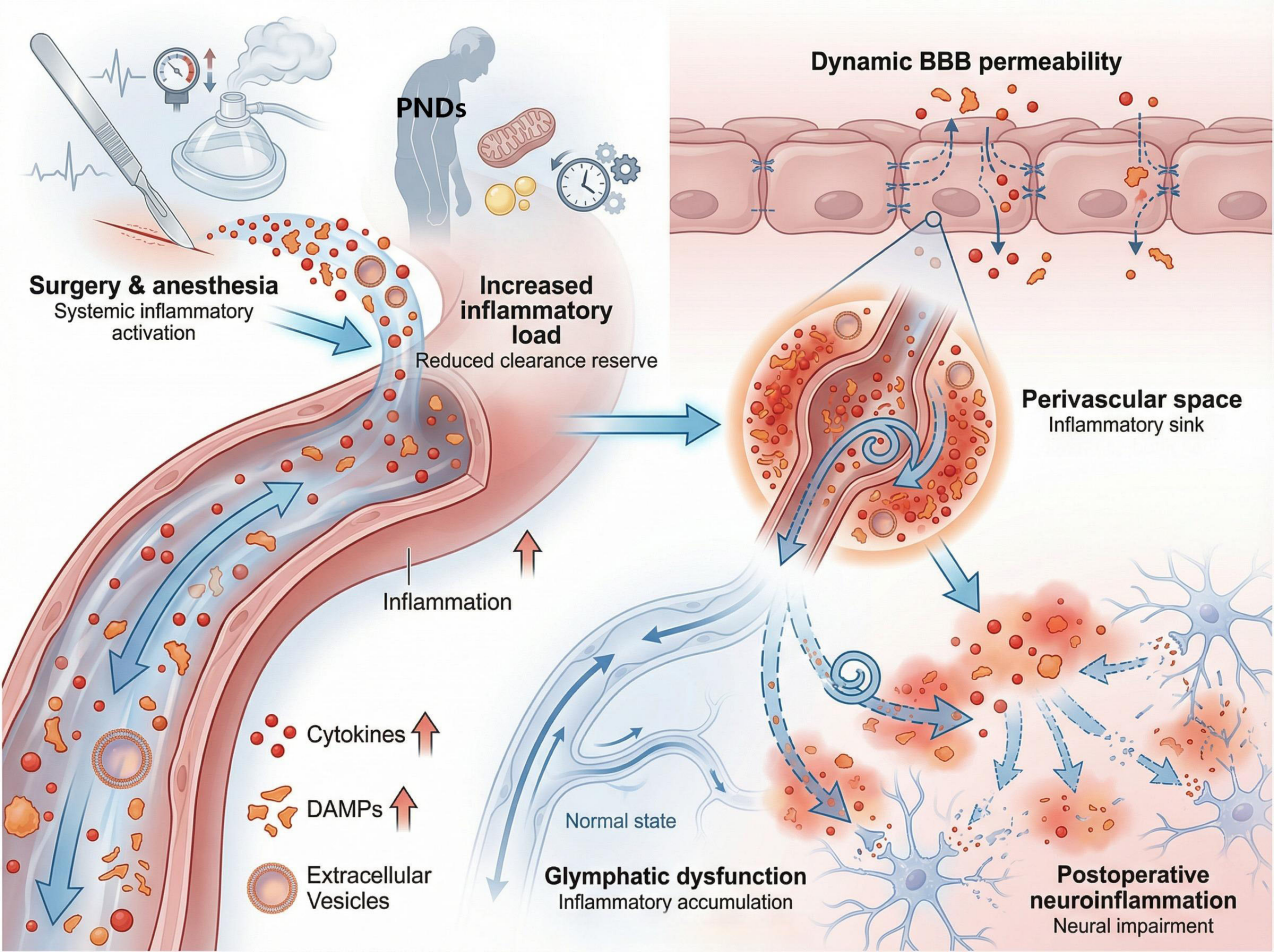
Schematic of the mechanism by which dysregulation of BBB–glymphatic–mLV system coupling promotes PND development. Disruption of BBB integrity, including impaired endothelial transport, a loss of AQP4 polarity, or weakened vascular dynamics, compromises glymphatic–mLV coupling, leading to the retention of solutes and inflammatory mediators; this feedforward loop of inadequate clearance and amplified neuroinflammation increases the risk of PNDs.

Integrating existing research facilitates the construction of a continuous mechanistic framework for PND development. Anesthesia and surgical stress first induce systemic inflammatory responses, promoting changes in BBB permeability and endothelial activation. This process increases the exposure to inflammatory factors at the blood–brain interface while altering the input environment and local fluid dynamics of the glymphatic–mLV system. Consequently, it amplifies neuroinflammation and dysregulates cognition-related brain regions. Notably, BBB dysfunction is not the sole determinant of PND development but represents a critical upstream link between inflammatory input and changes in the load on the clearance system of the brain. The specific role of the glymphatic–mLV system on this continuum, along with the differential effects of different surgical types on its structure and function, requires further elucidation through more systematic experimental and clinical studies (**Figure 3**). These topics will be discussed in the subsequent chapter.

## 4 The role of dysfunctional clearance by the glymphatic–mLV system in PNDs

### 4.1 Structural integration and functional characteristics of the glymphatic–mLV system

The glymphatic system originates from the subarachnoid CSF, enters the brain parenchyma via the PVS, and mediates solute transport through CSF–ISF exchange within the perivascular network (110, 111). In parallel, intramural periarterial drainage (IPAD) facilitates ISF efflux along the basement membranes of arterial smooth muscle cells, proceeding against blood flow toward the peripheral outflow pathway (110, 112). This clearance network operates across spatial scales—from perivascular CSF influx and parenchymal solute convection to periventricular ISF transport (113)—and is dynamically regulated by arterial pulsatility, respiratory rhythms, and sleep–wake states (114–116). mLVs are widely distributed throughout the dura mater region, where they connect to arachnoid villi to mediate CSF drainage while also establishing structural and functional links with the cranial bone marrow structure (117). Three structural types have been identified: lymphatic vessels closely accompanying blood vessels, independent lymphatic vessels situated away from blood vessels, and Lymphatic vessel endothelial hyaluronan receptor 1 (LYVE-1)-positive cell clusters located within vascular interstices (118). mLVs can be subdivided into several regions: dorsal pathways near the sagittal and transverse sinuses, which primarily facilitate CSF drainage and local immune regulation; basal pathways adjacent to the petrous and sigmoid sinuses, which serve as the core hub for CSF efflux; and ethmoid sinus mLVs in the ethmoid plate region, which may mediate drainage toward the nasal cavity (13). By receiving reflux from the subarachnoid space and PVS, mLVs transport solubilized proteins, extracellular vesicles, and immune cells into the peripheral lymphatic system, forming a crucial conduit for communication between the central and peripheral immune systems (119).

The glymphatic system concurrently facilitates material transport and metabolic clearance functions. It delivers glucose, lipids, amino acids, and apolipoprotein E to the brain parenchyma while regulating astrocytic lipid metabolism and signal transduction (120–122). Additionally, it is responsible for clearing lactic acid, soluble proteins (including Aβ and tau), and various exogenous particles (14, 123, 124). Impairment of its clearance ability leads to the accumulation of toxic metabolites and inflammatory mediators within the brain, which is correlated with increased neuroinflammation and tissue damage (72, 125). However, the movement of solutes from the parenchyma alone is insufficient for complete clearance. Solutes exiting the brain via perivenous routes are absorbed by mLVs, forming a continuous pathway between the glymphatic system and the peripheral lymphatic drainage pathway (13). mLVs, primarily located along the dural venous sinuses, are characterized by the presence of lymphatic endothelial cells with discontinuous basement membranes and no smooth muscle coverage (46). This unique structure allows the uptake of macromolecules and immune cells (13).

In addition to its metabolic role, the glymphatic system is critical for immune surveillance and response within the CNS. mLVs facilitate the transport of brain-derived antigens, immune cells, and inflammatory signals between the CNS and the peripheral immune system (126). The system also enables the influx of peripheral cytokines into the brain, allowing immune signals to reach the CNS. Moreover, brain-derived immune cells, such as dendritic cells (DCs), effector T cells (Teffs), and regulatory T cells (Tregs), are exported via mLVs to peripheral lymphoid organs (127). This bidirectional exchange is essential for maintaining CNS immune homeostasis and coordinating adaptive immune responses. During aging, astrocytes undergo functional changes that exacerbate neuroinflammation. CCL21 signaling promotes the migration of aging astrocytes from the brain to peripheral tissues via the glymphatic system, reducing the accumulation of proinflammatory mediators in the CNS and thereby mitigating chronic inflammation and supporting brain function (128, 129). Dendritic cells (DCs) are essential for initiating adaptive immune responses. They migrate through mLVs to dcLNs, where they present brain-derived antigens to naïve T cells, thereby activating the adaptive immune response. Chemokines such as CCL21 and CCR7 regulate this type of migration, ensuring efficient antigen presentation and T-cell activation (105, 126). Teffs and Tregs play pivotal roles in CNS immune responses (130, 131). In perioperative settings, Teff activation recruits these cells to the CNS, promoting neuroinflammation through the release of proinflammatory cytokines such as IFN-γ and TNF-α (132). These cytokines, in turn, activate microglia and astrocytes, amplifying the inflammatory response. In contrast, Tregs migrate to the CNS through the glymphatic system, where they suppress inflammation, promote immune tolerance, and facilitate tissue repair. The balance between Teffs and Tregs is critical for controlling neuroinflammation and supporting recovery following surgery or injury. Chemokines, including CCL2, CXCL10, and CCL5, regulate the migration of immune cells within the CNS (133, 134). CCL2 and CCR2 mediate the recruitment of monocytes and microglia to sites of inflammation, whereas CXCL10 and CXCR3 guide the migration of T cells to inflamed regions (135, 136). These chemokine–receptor interactions ensure the precise regulation of immune cell recruitment, shaping the intensity and resolution of neuroinflammatory responses.

In summary, the glymphatic–mLV system represents a continuous clearance pathway that not only transports metabolic byproducts but also maintains both central metabolic and immune homeostasis (13). Perioperative stress may impair this system, compromising its clearance efficiency and immune regulation and thereby increasing the risk of PNDs.

### 4.2 Impairments in the function of the glymphatic–mLV system induced by noncranial surgery

Noncranial surgeries, such as abdominal and orthopedic procedures, have been reported to affect the function of the glymphatic–mLV system, which contributes to brain waste clearance. These impairments—which are driven by systemic inflammation, cerebral hemodynamic disturbances, and alterations in astrocyte polarity—increase susceptibility to PNDs. However, the mechanisms of damage and the degree of disruption to the glymphatic–mLV system vary considerably depending on the type of surgery, and an understanding of these differences is key to developing strategies to mitigate PND risk.

Abdominal surgery causes a significant disruption to the glymphatic–mLV system, with laparotomies generally causing more pronounced effects compared to laparoscopic procedures. This effect is likely due to the more extensive mechanical trauma and inflammation caused by open surgeries. In 2024, Zhu et al. demonstrated that laparotomy upregulates MMP-9, which compromises both the BBB and AQP4 polarity, thus leading to a weakening of the glial–vascular interface and impairing glymphatic function (25). Moreover, in 2026, Feng et al. investigated aged mice after laparotomy and further demonstrated that glymphatic system dysfunction and AQP4 translocation were key pathological changes associated with perioperative cognitive impairment (137). These changes are thought to be more severe in open surgeries, as they involve more direct tissue manipulation, which induces stronger inflammatory responses compared to minimally invasive laparoscopic procedures. Although laparoscopic surgery also disrupts glymphatic function, the effect is generally milder, which is likely due to reduced tissue damage and inflammation. In 2024, Roy et al. used diffusion tensor imaging (DTI) to demonstrate that laparoscopic abdominal surgery in patients with obstructive sleep apnea (OSA) resulted in deterioration of the CSF clearance system, which was linked to long-term cognitive impairment (41). Although the laparoscopic approach is less invasive, it still has the potential to disrupt glymphatic–mLV function, thus suggesting that even minimally invasive surgeries can significantly impact brain clearance pathways.

Furthermore, preexisting limitations in brain clearance pathways—such as those observed in aging—significantly amplify the adverse effects of these surgeries. In 2025, Chen et al. reported that isoflurane anesthesia combined with abdominal surgery selectively reduced the activity of the glymphatic–mLV system in aged mice, with the degree of reduction being strongly correlated with impaired postoperative T-maze performance (138). However, these deficits could be mitigated by adeno-associated virus (AAV)-VEGF-C-mediated generation of mLVs, thus suggesting that the restoration of lymphatic drainage function could improve cognitive outcomes. This scenario highlights the importance of considering the individual patient’s baseline clearance function when assessing the potential impact of surgery on brain health.

Orthopedic surgery (which is exemplified by tibial fractures under sevoflurane anesthesia) also impairs the glymphatic–mLV system; however, the underlying mechanisms are somewhat different. Orthopedic trauma is driven by acute injury and the activation of localized neuroinflammatory pathways. In 2025, Yu et al. further demonstrated that tibial fractures induced under sevoflurane anesthesia disrupted the morphology and drainage capacity of mLVs, which was accompanied by impaired recognition and spatial memory (139). The mechanism of this effect is thought to involve the activation of the HMGB1/TLR4/NF-κB pathway, which exacerbates AQP4 depolarization and further impairs the glymphatic–mLV system (140). Unlike abdominal surgery—which affects a broader range of system components—orthopedic surgery seems to primarily impact the structural integrity of mLVs, thus suggesting that the damage is more localized and specific to lymphatic drainage function rather than the broader BBB or glial-vascular interface.

Thoracic and cardiac surgeries potentially exert the most severe impacts; however, these effects continue to be the least understood in terms of direct glymphatic evidence. These procedures combine intense systemic inflammation with profound cerebral hemodynamic changes, especially in cardiac surgeries involving extracorporeal circulation, which alters the arterial pulsatility that drives glymphatic flow (141–145). Although these factors theoretically suggest a greater magnitude of disruption compared to abdominal or orthopedic models, direct mechanistic studies specifically measuring mLV drainage in this context are currently limited. Further research is necessary to confirm whether this heightened physiological stress translates to a proportionately greater failure of brain clearance pathways.

Collectively, these findings suggest that noncranial surgery may increase the risk of PNDs by suppressing brain clearance pathways, intensifying intracerebral inflammation, and promoting metabolite retention (**Table 1**).

**Table 1:** Stratified comparison of glymphatic–mLV system impairment across noncranial surgical types relevant to PNDs.

Surgical type	Subtype	Population	Core mechanism of glymphatic–mLV system damage	Impact on glymphatic–mLV function	Associated cognitive phenotype	Reference
Abdominal Surgery	Laparoscopic Abdominal Surgery	OSA Patients	Perioperative inflammation and anesthesia further impair already compromised glymphatic clearance	Mild–Moderate	POCD	(41)
	Laparotomy	Aged Mice (18 months)	↑MMP-9 → dystroglycan cleavage → AQP4 depolarization → impaired glymphatic clearance	Severe	POCD	(25)
		Aged Mice (18 months)	Surgery delays CSF influx and hinders waste clearance	Severe	Postoperative Delirium	(138)
		Aged Mice (18 months)	Glymphatic system dysfunction and AQP4 translocation, accompanied by neuroinflammation and neuronal loss	Severe	PND	(137)
		Middle-aged Mice (dcLNs impairment)	Preexisting lymphatic impairment → worsened glymphatic dysfunction → A1 astrocyte activation & AQP4 depolarization → neuroinflammation (↑TNF-α, IL-1β, IL-6, HMGB1/TLR4/NF-κB) → neuronal damage → impaired exploratory & spatial memory	Severe–Critical	PND	(140)
Orthopedic Surgery	Tibial Fracture Surgery	Aged Mice (18 months)	Anesthesia + surgery → impaired mLV morphology/function → reduced lymphatic drainage → microglial activation (↑Iba1, CD68) & neuroinflammation (↑IL-1β, TNF-α) → cognitive impairment	Severe	POCD	(139)

Anesthesia can interfere with CSF–ISF exchange by modulating neural activity, cerebral hemodynamics, and respiratory rhythms, thereby altering the clearance of central inflammatory metabolites. Multiple studies have indicated that both general anesthesia and regional anesthesia can alter glymphatic kinetics by disrupting CSF regulatory mechanisms, such as arterial pulsation, respiratory drive, and neural activity coupling (146). Anesthesia-related decreases in blood pressure and heart rate are believed to impair CSF return efficiency, whereas alterations in sympathetic tone may further affect overall CSF circulation by suppressing CSF production (114, 138, 147, 148). Certain anesthetics, such as isoflurane and ketamine, can increase CSF production under specific conditions, exerting complex and context-dependent effects on CSF dynamics (149).

*In vivo* two-photon imaging studies have revealed that Ca²^+^ signaling within astrocytes is closely associated with the selective transport of small lipid-soluble molecules and the rapid propulsion of CSF within the glymphatic–mLV system (120). Fluid shear stress generated by perivascular CSF or interstitial cerebrospinal fluid flow mechanically activates NMDA receptors on astrocytes, inducing Ca²^+^ influx and thus regulating glymphatic transport efficiency (150). Ketamine may disrupt this mechanosensory regulatory mechanism by inhibiting these receptors and attenuating associated signaling pathways, thereby affecting CSF flow and solute clearance (151). Sevoflurane can also disrupt glymphatic transport at both the structural and functional levels by downregulating the expression of actin-binding proteins, weakening astrocytic Ca²^+^ currents, and inducing morphological remodeling (152, 153).

At the molecular level, astrocytic endfoot water and ion transport contributes to convection-driven CSF transport (154). Isoflurane inhibits Kir4.1/5.1 channel conductance and disrupts perivascular transport organization, thereby decreasing glymphatic transport efficiency (155). Conversely, mice anesthetized with 2.5% sevoflurane for 2 hours exhibit increased CSF flow, thus suggesting dose-dependent and directionally divergent modulation of the clearance system by different anesthetics (49). Notably, altered astrocyte endfoot transport properties are closely correlated with impaired glymphatic–mLV-mediated clearance and cognitive dysfunction, thereby potentially constituting a key cellular basis for anesthesia-related neuroplasticity (156).

### 4.3 Damage to the glymphatic–mLV system induced by an intracranial structural disruption

Compared with noncranial surgeries, intracranial procedures, trauma, and skull defects directly disrupt perivascular pathways and mLV drainage through acute alterations in intracranial pressure, brain compliance, and CSF dynamics, resulting in structural changes in the clearance system of the brain. Imaging and clinical studies further substantiate the persistent effect of structural disruptions on clearance systems. In 2024, Guo et al. used diffusion tensor imaging with DTI-ALPS and showed that patients with severe traumatic brain injury (TBI) exhibited persistent dysfunction of the glymphatic–mLV system following decompressive craniectomy, with a significant negative correlation between the skull defect area and the ipsilateral DTI-ALPS index (157). Intracranial space-occupying lesions or chronic structural alterations are also closely associated with reduced CSF clearance rates (158). In 2020, Hauglund et al. reported that chronic implantation of Electroencephalography (EEG) electrodes or cranial windows in mice elicits meningeal lymphangiogenesis and increases glymphatic CSF influx, suggesting that CNS clearance pathways undergo adaptive remodeling in response to sustained mechanical stimulation (159).

More compelling causal evidence arises from the direct manipulation of mLVs. As the principal efflux pathway for CSF, its macromolecular solutes, and immune cells exiting the cranial cavity, mLVs establish a continuous drainage route to the peripheral immune system via dcLNs and submandibular lymphatic structures (16, 118, 160). Direct disruption of mLVs significantly reduces the clearance of pathological proteins such as Aβ, amplifies neuroinflammatory responses, and results in cognitive deficits (161). Downstream obstruction models further substantiate this causal relationship. The ligation of dcLNs effectively blocks mLV outflow, leading to the intracerebral accumulation of Aβ and tau, which is accompanied by increased neuroinflammation, reduced levels of synapse-associated proteins, impaired AQP4 polarization, and diminished cognitive and exploratory behaviors (22, 85).

**Figure 4:**
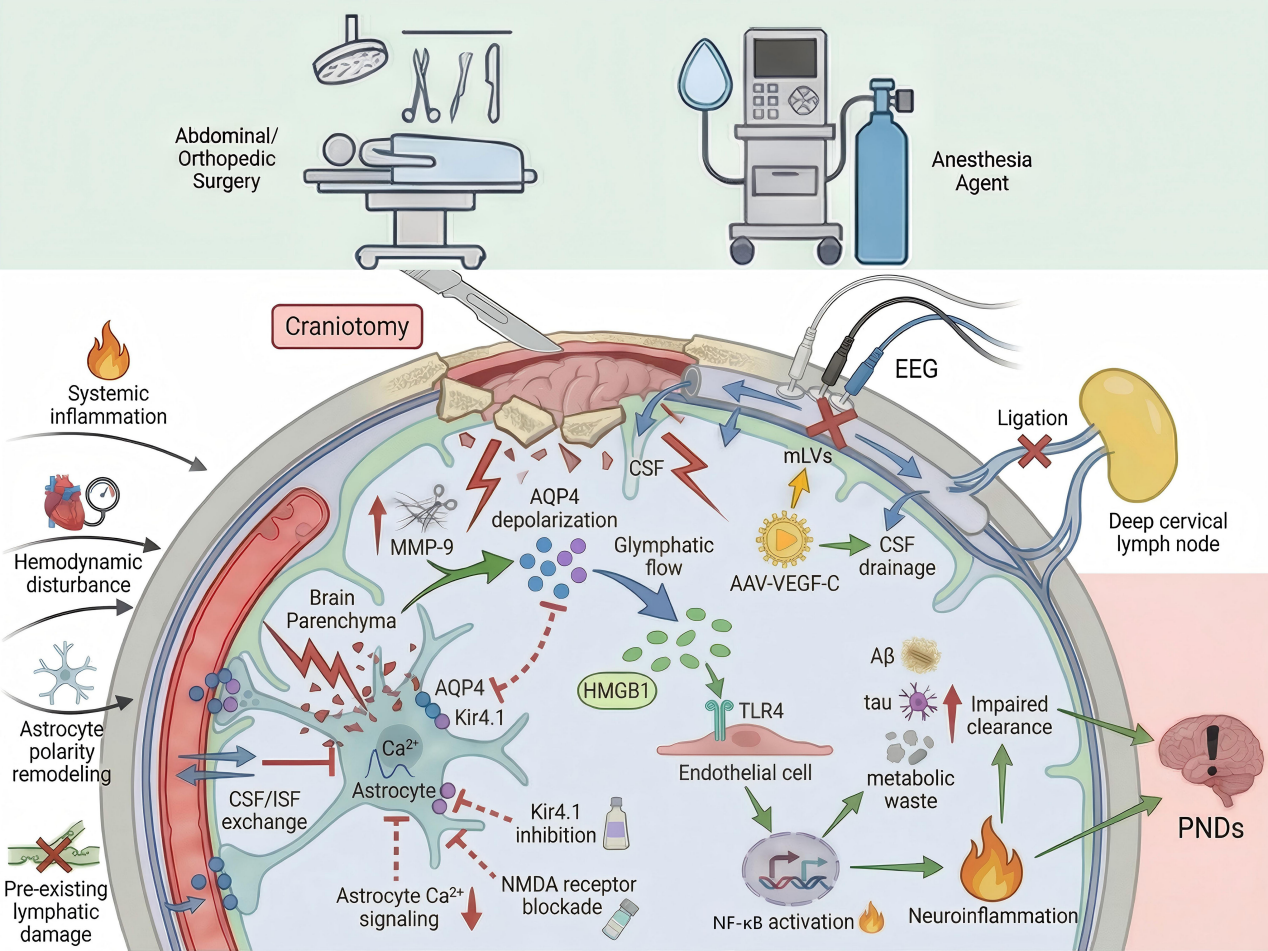
Perioperative dysregulation and structural disruption of the glymphatic–mLV system. Noncranial surgery and anesthesia induce systemic inflammation, hemodynamic disturbances, and changes in astrocyte polarity, leading to AQP4 depolarization, Kir4.1 inhibition, and disrupted Ca²^+^ signaling. These changes impair CSF–ISF exchange and glymphatic–mLV drainage; promote the retention of Aβ, tau, and inflammatory mediators; and are associated with the activation of HMGB1/TLR4/NF-κB-related inflammatory pathways in experimental settings. In contrast, intracranial structural disruption or downstream lymphatic obstruction directly alters intracranial pressure, brain compliance, and CSF dynamics, mechanically compromising glymphatic–mLV integrity and efflux. The resulting persistent clearance failure further increases susceptibility to PNDs.

Collectively, these findings indicate that dysfunction of the glymphatic–mLV system alone is sufficient to compromise central clearance efficiency and may increase vulnerability to PNDs in the context of surgical manipulation (**Figure 4**).

### 4.4 Glymphatic–mLV system dysfunction in other neurological disorders and its association with PNDs

Increasing evidence indicates that dysfunction of the glymphatic–mLV system is not unique to PNDs but represents a convergent pathophysiological feature across multiple neurological disorders. It also manifests in various neurological disorders, including Alzheimer disease (AD), Parkinson disease (PD), TBI, stroke, multiple sclerosis (MS), cancer-related cognitive impairment (CRCI), and chronic vascular or metabolic encephalopathy (13, 112, 124, 162–167). Despite their highly heterogeneous etiologies and temporal progressions, these disorders share a common feature: an impaired solute clearance capacity within the brain or a kinetic imbalance between influx and efflux.

In acute injuries, such as TBI or stroke, glymphatic–mLV clearance functions are often rapidly suppressed, leading to the acute retention of inflammatory mediators and metabolic byproducts (166, 168, 169). In chronic neurodegenerative diseases, including AD and PD, this suppression gradually evolves into structural degeneration and persistent clearance insufficiency (148, 164, 170). PNDs likely occupy an intermediate position on this continuum; a significant, transient increase in the perioperative inflammatory load coupled with a relatively insufficient baseline clearance capacity can result in the retention of metabolic byproducts and inflammatory factors, thereby impairing neural function. Multiple studies suggest overlapping pathologies between PNDs and AD, including detectable Aβ/tau accumulation in the brain parenchyma and elevated levels of related proteins in the CSF, which lead to neuroinflammation and neuronal damage (171–173).

Perioperative peripheral inflammation and anesthetic stimulation jointly disrupt central immune homeostasis, destabilizing microglial polarization and promoting a shift toward the M1 phenotype (174). M1 microglia induce the generation of neurotoxic A1-type astrocytes through the secretion of signaling molecules such as IL-1β, iNOS, and complement C1q (175). These astrocytes produce additional inflammatory mediators, amplifying local inflammatory responses and increasing the risk of structural and functional neuronal damage (35, 84, 176). During this process, reactive astrocytes redistribute AQP4 from perivascular regions to cell bodies or nonperivascular areas. This redistribution may disrupt the kinetics of CSF–ISF exchange in the brain, thereby reducing the overall clearance efficiency of the glymphatic–mLV system. Such disruptions may contribute to the onset and persistence of symptoms in PNDs (85, 98, 177).

Many studies have investigated the role of the glymphatic–mLV system in PNDs. Given the varying strengths and types of evidence across these studies, we present a hierarchical framework to systematically summarize and organize the current knowledge (**Table 2**).

**Table 2:** Hierarchical framework of evidence for glymphatic–mLV system involvement in PNDs.

Evidence level	Evidence type	Core supporting points
I	Evidence from mechanistic interventions in perioperative settings	Targeted modulation of lymphoid and meningeal lymphatic function in surgical or anesthetic models accompanied by cognitive assessments suggests that clearance pathways functionally regulate perioperative neurocognitive alterations (24–26, 41, 138–140, 159).
II	Evidence supporting peripheral–central coupling	The glymphatic–mLV system structurally and dynamically links the peripheral immune environment with the central microenvironment, providing a potential pathway for peripheral inflammatory signals to influence the brain microenvironment (47, 110, 160, 292, 293, 298).
III	Evidence supporting the clearance system structure	Structural remodeling and loss of molecular polarity within the glymphatic–mLV axis constitute the foundational framework for impaired fluid transport and metabolic clearance (45, 110, 117, 118).
IV	Evidence for clinical relevance	The perioperative inflammatory burden or clearance-related markers exhibit clinically relevant associations with cognitive outcomes (5, 246, 247, 294, 295).
V	Cross-disease extrapolation and hypothesis-supporting evidence	Analogous studies in other neurological diseases or models that link clearance dysfunction to neurofunctional alterations support the extrapolation of the mechanisms (112, 164, 166–168, 172, 302).

## 5 Other peripheral inflammatory pathways and their interactions with glymphatic–mLV clearance in PNDs

In addition to the BBB and glymphatic–mLV system, the systemic inflammatory response induced by surgical stress can influence the central nervous system through multiple peripheral-to-central routes, including the blood–cerebrospinal fluid barrier (BCSFB), circumventricular organs (CVOs), and vagal afferent pathways (13, 178). In the context of PNDs, the mechanistic importance of these routes is based not only on the transmission of inflammatory signals to the brain but also on their synergistic or antagonistic interactions with glymphatic–mLV-mediated clearance (depending on whether the brain clearance reserve is preserved or overwhelmed) (179–181).

The BCSFB, which is primarily composed of the choroid plexus (CP), serves as a critical interface between the circulatory system and the central nervous system (182). The CP consists of tightly connected epithelial cells and fenestrated capillaries that produce CSF while actively regulating its composition and turnover through selective transport processes (183). Under physiological conditions, the choroid plexus supports central homeostasis by delivering micronutrients and neurotrophic factors via the CSF and may act synergistically with glymphatic–mLV clearance by maintaining a solute milieu that is conducive to downstream transport and resolution (184–186). However, during perioperative inflammation, the disruption of CP secretory homeostasis increases the release of chemokines, proteins, and other inflammatory mediators into the CSF and may facilitate limited leukocyte trafficking (187, 188). Furthermore, inflammatory alterations in the CSF composition may disturb fluid dynamics and reduce clearance efficiency (189, 190). Under these conditions, the relationship between the BCSFB and glymphatic–mLV system may shift from physiological synergy to functional antagonism, as excessive intrathecal inflammatory input increases the burden on downstream clearance pathways and hinders the resolution of central inflammation (191–193).

CVOs are located around the third and fourth ventricles and are characterized by highly vascularized structures that lack a typical BBB (194). In CVOs, neurons and glial cells extensively express cytokine and metabolic signaling receptors, thus facilitating the direct sensing of circulating inflammatory signals (195, 196). These signals mediate central responses through downstream neural circuits or via CSF-mediated information exchange (197, 198). Functionally, CVOs are coupled to the glymphatic–mLV system because the inflammatory signals that they relay still require subsequent dispersion and clearance within the central compartment (181, 199). When inflammatory input is limited and clearance function is intact, this coupling effect may be regarded as synergistic, thus allowing for immune sensing to be followed by timely signal resolution (200). However, under perioperative stress, excessive CVO activation combined with impaired glymphatic–mLV flow may prolong local cytokine exposure and solute retention, thereby creating a functionally antagonistic interaction that amplifies the mismatch between inflammatory input and clearance output (161, 201).

Furthermore, vagal afferent fibers form a rapid neural pathway that transmits immune and metabolic signals from the periphery to the nucleus tractus solitarius and higher integrative centers such as the hypothalamus (202). This pathway triggers central inflammation-related responses, which subsequently impact postoperative neurocognitive outcomes (180, 203, 204). Vagal afferent activation also modulates neural activity in the brainstem and limbic system, thereby altering cerebral blood flow, neurovascular coupling, and CSF dynamics, all of which are closely tied to glymphatic function (205, 206). In addition, vagally mediated changes in norepinephrine signaling may influence microglial reactivity and lymphatic vessel contractility, thereby indirectly affecting meningeal lymphatic drainage (207, 208). Under physiological or moderate stimulation, such regulation may act synergistically with glymphatic–mLV function by maintaining the neural and vascular conditions required for effective clearance (209, 210). In contrast, excessive or prolonged vagal activation during perioperative inflammation may perturb these same conditions, thereby producing a functionally antagonistic effect that limits clearance efficiency and sustains central immune activation (170).

Taken together, the BCSFB, CVO, and vagal afferent pathways do not simply provide parallel routes of inflammatory signaling to the brain. Rather, they converge on the glymphatic–mLV system at different levels; specifically, the BCSFB modulates the inflammatory burden within the CSF compartment, CVOs amplify central inflammatory sensing, and vagal afferents reshape the neural and vascular dynamics that are required for effective transport (211–213). Under physiological conditions, these routes may act synergistically with glymphatic–mLV clearance to support signal integration and homeostatic resolution (19, 214). However, during perioperative stress (when inflammatory input exceeds the baseline clearance reserve), these interactions may become functionally antagonistic, thus promoting solute retention, sustained microglial activation, prolonged neuroinflammation, and the ultimate development of PNDs.

## 6 Methods for assessing glymphatic–mLV system function during the perioperative period

Perioperative glymphatic–mLV function is highly dynamic, with structural integrity, CSF–ISF exchange, molecular transport, and systemic functional outcomes often occurring asynchronously. No single imaging or molecular marker can fully capture the functional state of this system. Animal imaging, human radiology, genomics, molecular tracers, and neurofunctional readouts each provide complementary insights across different scales. A multimodal, integrative framework is therefore needed to comprehensively evaluate perioperative glymphatic–mLV-mediated clearance and its systemic consequences.

### 6.1 Structural and dynamic imaging of the glymphatic–mLV system in animal models

In rodent models, multiphoton microscopy is widely used for the real-time tracking of CSF flow along perivascular spaces and its exchange with brain interstitial fluid, providing excellent temporal and spatial resolution. However, its limited field of view and invasive nature hinder its clinical translation (14, 215). Transcranial macroscopic fluorescence imaging enables quantitative whole-brain CSF flow comparisons in mice and large animals, despite its limited spatial resolution (215, 216). Recent advances in whole-brain clearing techniques (CUBIC and iDISCO) combined with light-sheet fluorescence microscopy (LSFM) have allowed high-resolution mapping of mLVs, whereas photoacoustic imaging provides real-time monitoring of vascular and lymphatic dysfunction during perioperative interventions (18, 217–220).

### 6.2 Human imaging assessments

#### 6.2.1 Contrast-enhanced kinetic studies

Dynamic contrast-enhanced MRI (DCE-MRI), which employs gadolinium-based contrast agents, enables the dynamic tracking of CSF distribution and clearance across the entire brain (221–224). Advances in real-time and ultrafast MRI techniques have improved temporal resolution, making clearance kinetics increasingly assessable (225, 226). However, the trade-off between temporal resolution and spatial resolution and the potential risks associated with the use of gadolinium-based agents in perioperative patients require cautious application (227, 228).

#### 6.2.2 Alternative noncontrast measures

Noncontrast techniques such as diffusion tensor imaging (DTI) and phase-contrast MRI (PC-MRI) are valuable alternatives. DTI can be used to indirectly assess lymphatic pathways using diffusion anisotropy, whereas PC-MRI can be used to directly measure CSF pulsation velocities, offering more accurate data on cerebrospinal circulation (229–231). Additionally, 7-T ultrahigh-field MRI with black-blood sequences allows direct visualization of mLVs, providing anatomical evidence of lymphatic dilation following postoperative inflammation. This high-resolution imaging technique is crucial for understanding mLV structural changes after surgery and offers a novel clinical tool (232–234). T2-weighted fluid-attenuated inversion recovery (FLAIR) and T1 black-blood MRI also enables the visualization of mLVs postcontrast, supporting *in vivo* evidence of structural alterations (235–237). These techniques form an anatomical basis for exploring perioperative inflammatory signaling between mLVs and the peripheral immune system, although functional assessments still rely on indirect inference. During the perioperative period, near-infrared spectroscopy (NIRS) provides repeatable bedside measurements, facilitating real-time clinical monitoring of clearance, despite limitations in spatial resolution and pathway specificity (238, 239).

#### 6.2.3 Positron emission molecular tracer imaging

Positron emission tomography (PET) and single-photon emission computed tomography (SPECT) provide unique insights into the evaluation of molecular exchange between the CSF, ISF, and peripheral compartments. Despite its higher costs, limited spatial resolution, and constraints related to radionuclide half-lives, dynamic SPECT is a highly sensitive approach for assessing regional tracer exchange and peripheral clearance, thereby providing a more direct reflection of molecular-level transport efficiency. These nuclear medicine techniques have been utilized across various neurological disorders to assess the state of material exchange between CSF, the central nervous system, and peripheral tissues (240–243).

### 6.3 High-throughput genomics and immune profiling of the glymphatic–mLV system

Advances in high-throughput genomics have provided crucial insights into the molecular dynamics of the glymphatic–mLV system, particularly during the perioperative period. Single-cell RNA sequencing (scRNA-seq) and single-nucleus RNA sequencing (snRNA-seq) enable the identification of immune cell subsets and their dynamic changes, allowing for the tracking of immune responses within these systems over time. For instance, van Hove et al. utilized scRNA-seq to map the diversity of brain-resident macrophages, including border-associated macrophages (BAMs), in the dura mater and choroid plexus, shedding light on the immune landscape of the brain (244). Spatial transcriptomic methods, such as Visium and MERFISH, offer the potential to map immune-vascular interactions within the meningeal compartment. However, the application of these technologies to meningeal immunity is still in its early stages and is limited by challenges in spatial resolution and tissue-specific constraints. Mass cytometry (e.g., CyTOF) further enhances the analysis of meningeal macrophage phenotypes by enabling high-dimensional immune profiling. In 2023, Quintana et al. integrated CyTOF with transcriptomics to explore the immune response during chronic Trypanosoma brucei infection, revealing ectopic lymphoid structures and autoreactive B cells, thus highlighting the utility of these methods for studying the glymphatic–mLV system (245).

At the molecular level, perioperative biomarkers such as S100 calcium-binding protein β (S100β), Glial fibrillary acidic protein (GFAP), and Neurofilament light chain (NfL), which are elevated in CSF and plasma, serve as indicators of neural injury and impaired clearance functions, particularly in patients with POCD (246–248). Phosphorylated tau (p-tau) is a key marker of disrupted clearance mechanisms and neurodegeneration, whereas soluble TREM2 (sTREM2) has been identified as a biomarker for glial activation, particularly in postoperative delirium, linking immune activation to cognitive dysfunction (249).

Neurofunctional monitoring techniques such as EEG and Magnetoencephalography (MEG) also offer valuable insights into perioperative neurological changes. Altered neural oscillations, particularly a reduction in gamma rhythm, reflect disruptions in neuronal synchronization and network stability, which are often linked to the accumulation of metabolic waste products such as Aβ and HMGB1 (159, 250, 251).

**Figure 5:**
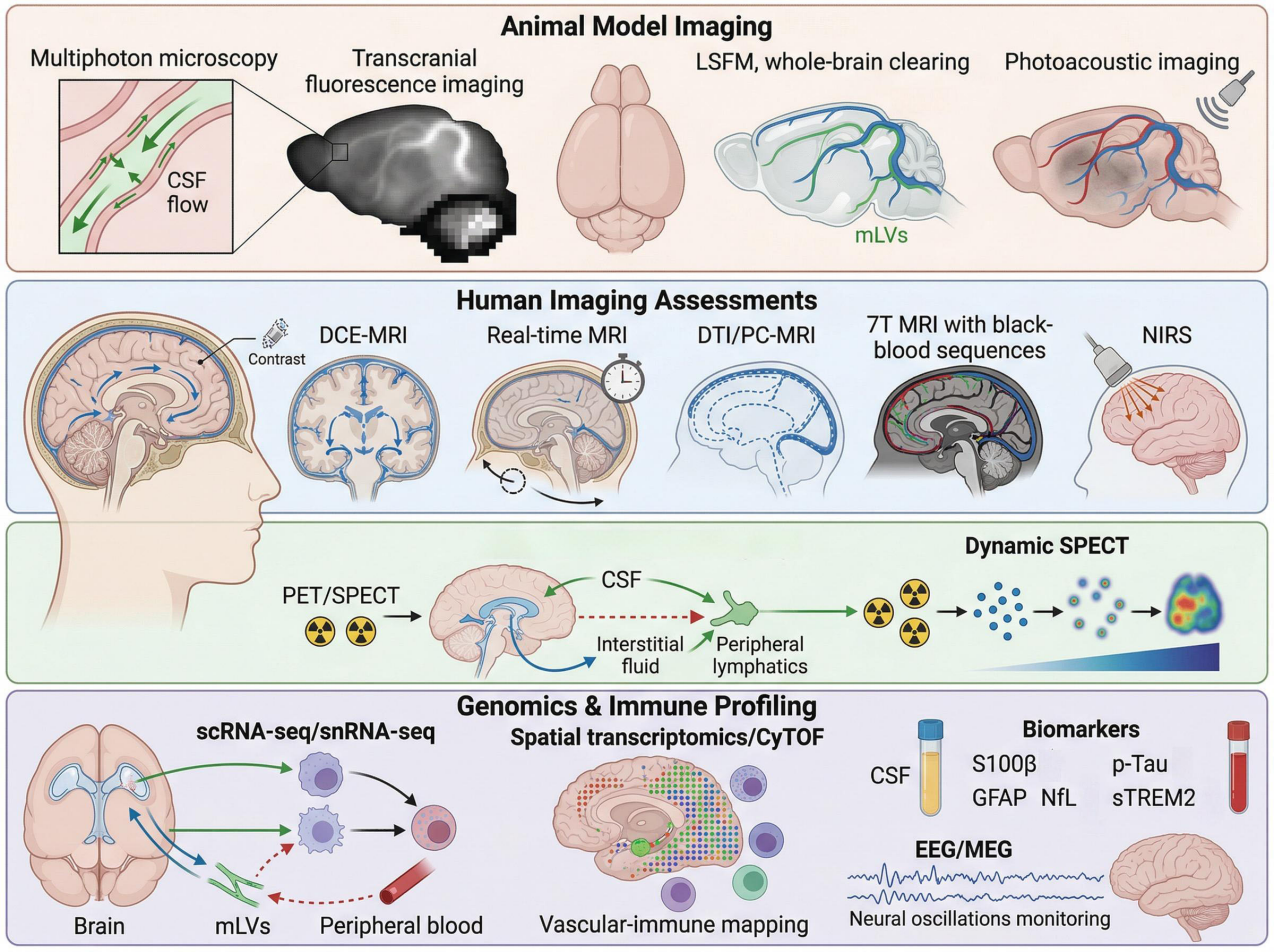
Multilevel framework for evaluating perioperative glymphatic–mLV system function. Imaging and molecular techniques for assessing glymphatic–mLV system function during the perioperative period include structural and dynamic imaging in animal models (multiphoton microscopy, transcranial fluorescence, LSFM, and photoacoustic imaging) and human assessments (DCE-MRI, DTI, PC-MRI, and 7-T MRI). Molecular tracer imaging (PET and SPECT) can be used to track CSF–ISF exchange, whereas high-throughput genomic methods (scRNA-seq, snRNA-seq, spatial transcriptomics, CyTOF) can provide insights into immune dynamics. Biomarkers (S100β, GFAP, NfL, p-tau, and sTREM2) and EEG/MEG can be used to detect neural injury, impaired clearance, and neurodegeneration.

Current techniques provide structural, kinetic, or systemic functional information, but direct correspondence across modalities remains unclear. Multimodal integration can help capture complementary aspects of the function of this system. Future studies should aim to establish longitudinally coupled and standardized measures to inform perioperative risk evaluations (**Figure 5**).

## 7 Functional regulation and clinical translation of the glymphatic–mLV system during the perioperative period

Perioperative cerebral clearance may be influenced by multiple interrelated approaches, including the modulation of central clearance by anesthetic state, the perioperative optimization of basic physiological parameters, and metabolic–immunological interventions that reduce the burden on clearance systems. These strategies can affect glymphatic–mLV function, potentially supporting waste removal and contributing to a lower risk of PNDs. Notably, the functional recovery of the clearance pathway evolves across postoperative stages, creating periods of relative vulnerability that may guide temporally staged interventions and translational applications.

### 7.1 Anesthetic modulation of central clearance dynamics

#### 7.1.1 Noradrenergic tension and slow-wave-like brain states

Ketamine–xylazine (KX) was among the earliest anesthetic combinations investigated in relation to the glymphatic system; its effects on cerebrospinal and ISF dynamics have been studied but remain context dependent (14). Early studies suggested increased solute clearance in the extracellular space of KX-anesthetized rats compared with pentobarbital-anesthetized rats (252). Subsequent studies indicated that KX may augment glymphatic–mLV system function, potentially through the amplification of slow-wave oscillations and a reduction in heart rate (250). High-resolution 3D FISP-MRI revealed greater CSF flux and a greater flow velocity in KX-anesthetized mice than in isoflurane-anesthetized mice (147). Under KX anesthesia, CSF contrast agents administered to mice diffuse along the circle of Willis, with some permeating the brain parenchyma via perivascular spaces and a substantial portion circulating through the CSF system, including routes via the nasal turbinate and pharyngeal lymphatics. In contrast, under isoflurane anesthesia, most CSF flows toward the spinal cord and cranial ganglia rather than entering the brain parenchyma (147). However, some studies comparing KX anesthesia with the awake state did not observe an increase in glymphatic–mLV system function (253).

Dexmedetomidine (Dex) is a highly selective α_2_ adrenergic receptor agonist that induces a sleep-like state in the brain by inhibiting the locus coeruleus–noradrenergic system, which reduces central norepinephrine levels and induces slow-wave-like electroencephalographic activity (116, 254). Animal studies have indicated that this state increases the exchange of CSF and ISF, improves glymphatic–mLV system function, and promotes distribution within the brain following CSF-based administration. Additionally, improved long-term cognitive outcomes have been observed in certain developmental models (26, 255, 256). Imaging studies further corroborate this trend. In 2017, Benveniste et al. reported that under anesthesia with Dex combined with low-dose isoflurane, the efficiency of whole-brain solute transport was greater than that under anesthesia with high-dose isoflurane, with an accelerated trend in contrast agent clearance in the hippocampal region (257). In 2021, Ozturk et al. reported that Dex combined with low-dose isoflurane primarily expanded the basal cisterns and periarterial CSF spaces, whereas high-dose isoflurane caused mild parenchymal swelling of the brain (258). Although glymphatic flow was not directly measured in this study, the observed differences in the CSF distribution suggest that varying depths of anesthesia may affect CSF–ISF exchange efficiency by modulating glymphatic–mLV pathways. In clinical research, Dex is considered to potentially reduce the risk of POD in elderly patients (259). An early meta-analysis revealed that its preventive effect was more pronounced in patients who underwent cardiac surgery (260). However, a subsequent updated meta-analysis did not consistently reproduce these findings. The discrepancies in the evidence suggest that the neuroprotective effects of Dex may depend on the characteristics of specific patient populations and context of perioperative management. Additionally, adverse reactions such as bradycardia and hypotension necessitate careful assessment in high-risk patients (261).

#### 7.1.2 Inhalation and intravenous anesthesia and the glymphatic–mLV system

Under surgical stress, the adverse effects of isoflurane on glymphatic function are particularly pronounced in susceptible individuals (138). High-dose or prolonged exposure to isoflurane impairs glymphatic flow and exacerbates neuroinflammation, partly through MMP-9 upregulation and disruption of perivascular homeostasis (24, 25, 262). These effects are further aggravated in conditions of preexisting mLV dysfunction, suggesting that the baseline functional state of meningeal lymphatics may modulate the severity of inhalation anesthetic-related CNS injury (140). Notably, some imaging studies utilizing the long-term distribution of heme-based contrast agents have indicated reduced brain retention during anesthesia. However, this phenomenon likely reflects the combined regulation of substance transport pathways by the anesthetic state and circadian rhythms rather than merely indicating increased clearance (263).

In contrast, the effects of sevoflurane on the glymphatic–mLV system appear to be more modifiable. Recent imaging studies at the human level have directly shown that sevoflurane disrupts macroscopic CSF dynamics. Zimmermann et al. employed functional magnetic resonance imaging in healthy volunteers and reported that sevoflurane anesthesia significantly reduced the amplitude of CSF flow in the basal cisterns and disrupted global functional connectivity within gray matter, as well as the coupling between gray matter and CSF throughout the brain (144). In surgical models in elderly patients, sevoflurane inhibits the drainage of mLVs and restricts the clearance of inflammatory factors (139). However, this clearance impairment can be partially reversed by interventions that improve lymphatic drainage and perivascular exchange, indicating potential therapeutic strategies for inhalation anesthesia-related clearance deficits (26, 139).

Propofol, a widely used intravenous anesthetic in humans, exhibits both anti-inflammatory and neuroprotective properties (264, 265). Studies suggest that propofol enhances glymphatic transport (262). Additionally, pentobarbital has been shown to significantly enhance glymphatic transport in animal models, although benzodiazepines have largely been replaced in anesthesia for humans (250). Other anesthetics utilized in animal studies, such as chloroform and ether, inhibit CSF tracer transport along glymphatic–mLV pathways (250).

#### 7.1.3 Mixed anesthesia and state-dependent effects

The classical experimental evidence supporting the glymphatic system has predominantly been established under conditions of mixed anesthesia rather than the use of a single anesthetic agent. Direct comparisons of various anesthetic regimens have revealed that glymphatic outflow is most pronounced during ketamine/haloperidol anesthesia (258, 262). In contrast, isoflurane anesthesia alone suppresses glymphatic function, with this inhibitory effect being partially reversible when noradrenergic blockade is applied in conjunction with isoflurane anesthesia (250). However, the influence of mixed anesthesia on glymphatic-like function clearly depends on the dosage and specific conditions. MRI and near-infrared fluorescence tracer studies have indicated that, within certain dosage ranges, both isoflurane and KX anesthesia may inhibit CSF inflow into the brain parenchyma while facilitating CSF drainage through extracranial lymphatic pathways. These findings suggest that the anesthetic depth and associated physiological parameters significantly affect experimental outcomes (262). Consistent with these findings, *in vivo* microimaging evidence has shown that periarterial spaces surrounding superficial cerebral arteries create a low-resistance, pulsatile CSF flow pathway, which provides the necessary hydrodynamic foundation for CSF–ISF exchange under mixed anesthesia conditions. However, this result should not be interpreted as a direct increase in clearance efficiency. Moreover, in models utilizing ketamine/thiobarbiturate anesthesia, selectively enhancing or inhibiting the function of mLVs significantly alters Aβ clearance and the level of the immune response (161). This phenomenon indicates that under mixed anesthesia conditions, the effects of intracerebral clearance cannot be attributed to a single lymphatic pathway but may instead reflect a coupled regulatory relationship between the glymphatic system and mLV reflux.

**Figure 6:**
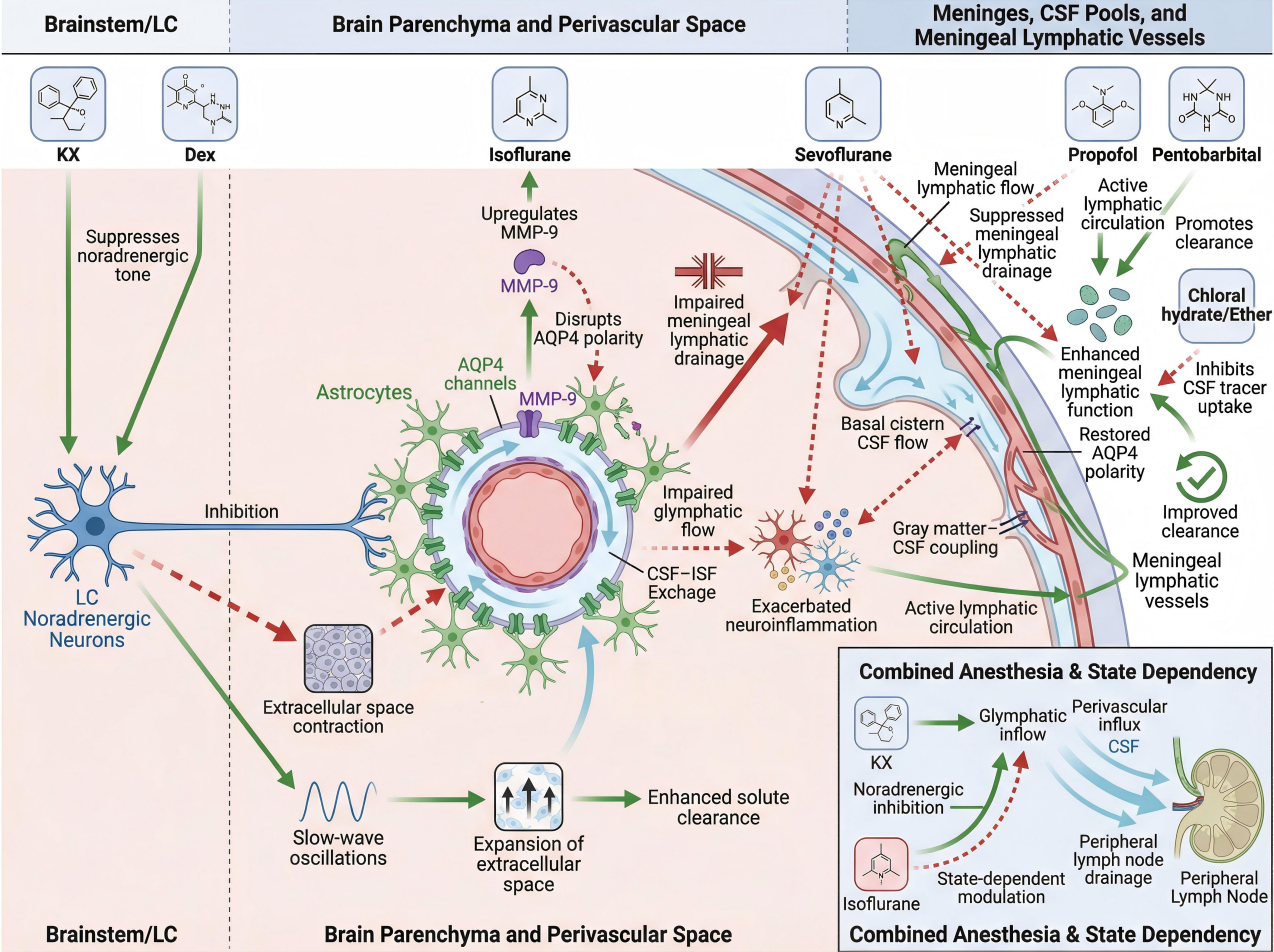
Regulatory effects of anesthesia on the central clearance microenvironment. Schematic of the effects of anesthetics on the locus coeruleus (LC), brain parenchyma, PVS, meninges, and glymphatic–mLV system. KX and Dex inhibit LC noradrenergic neurons, enhancing slow-wave oscillations and expanding PVS and ISF to promote solute clearance. Inhalation anesthetics (isoflurane and sevoflurane) alter MMP-9 levels and AQP4 polarity, impairing CSF–ISF exchange and mLV drainage and potentially exacerbating neuroinflammation. Intravenous anesthetics (propofol and pentobarbital) restore AQP4 polarity, increase lymphatic flow, and facilitate CSF/ISF solute clearance, whereas chloral hydrate and ether reduce CSF tracer uptake.

The anesthetic state itself may remodel CSF outflow pathways. Animal studies have indicated that under specific anesthetic conditions, CSF tends to flow more through perivascular spaces, while drainage rates to peripheral tissues and lymph nodes decrease. Conversely, in the awake state, CSF is primarily excreted via peripheral lymphatic pathways (266). Notably, these findings predominantly originate from specific combinations of anesthetics, and their applicability to other anesthetic regimens remains unclear. Research suggests that the depth of anesthesia may further modulate glymphatic–mLV system function; however, this state dependency requires validation under strictly controlled conditions (159) (**Figure 6**).

### 7.2 Physiological modulators of glymphatic–mLV-mediated clearance

Postural changes affect venous return, intracranial pressure, and PVS dynamics via the gravity-driven redistribution of blood and CSF, thereby modulating CSF–ISF exchange (267, 268). Studies have shown that the efficiency of transport and clearance within the glymphatic system is highest in the lateral decubitus position, intermediate in the supine position, and lowest in the prone position (113, 268, 269).

The glymphatic–mLV system is strongly dependent on the brain state, with enhanced function during sleep and suppression during wakefulness, in accordance with a stable circadian rhythm (270). Animal studies have indicated that tau protein and lactate levels in hippocampal CSF fluctuate during the sleep–wake cycle, increasing during periods of dominant neuronal activity and decreasing during the rest or sleep phases (271). This metabolic rhythm mirrors the temporal activity of glymphatic–mLV-mediated clearance, which is consistent with enhanced metabolite clearance during sleep (272). This temporal dependency involves multiple rhythmic regulatory components, including BMAL1-dependent circadian modulation of perivascular exchange, rhythmic variations in choroid plexus CSF production, and temporal differences in clearance efficiency across brain regions (273, 274). Thus, the sleep architecture and circadian rhythm not only shape the temporal organization of neuronal activity but also influence the central clearance of perioperative metabolic and inflammatory loads by defining operational windows for the glymphatic–mLV system.

Imaging studies have revealed a close correlation between respiratory phases and CSF flow: the inspiratory CSF velocity increases, whereas breath holding significantly suppresses its movement (275). Respiratory movements regulate CSF flow in the perivascular space by influencing venous return and fluctuations in intracranial pressure, thereby affecting the efficiency of CSF–ISF exchange (115). These findings suggest that respiratory patterns may represent a potential noninvasive intervention point for continuously modulating central clearance kinetics.

### 7.3 Metabolic and immunological background interventions: reducing the burden on clearance systems

At the physiological and metabolic levels, anti-inflammatory and immunomodulatory approaches may help to restore glymphatic–mLV function. Polyunsaturated fatty acids, AV-001, and β-hydroxybutyrate appear to mainly promote clearance recovery by reducing neuroinflammation and improving perivascular homeostasis (276, 277). In addition, digoxin may create a more favorable milieu for brain clearance by modulating immune–lymphatic coupling (278, 279). Trifluoperazine has also been observed to enhance postoperative solute clearance and mitigate PND-related alterations, thus further supporting the therapeutic potential of the targeting of the clearance system (280).

### 7.4 Targeted regulation and translational exploration of perioperative clearance pathways

Multiple noninvasive neuromodulations and physical interventions have the ability to remodel cerebral fluid dynamics. Notably, postoperative transcranial magnetic stimulation (TMS) has demonstrated a preliminary clinical benefit in elderly surgical patients, with improved memory performance on postoperative Day 7 and abbreviated mental test scores on Day 30 being observed, whereas parallel findings in aged PND mouse models suggest that these effects are associated with the restoration of glymphatic function and the attenuation of both neuroinflammation and neuronal loss (137). Low-intensity transcranial ultrasound enhances the whole-brain distribution of various small and macromolecular agents, and this process is associated with increased lymphatic pathway activity and improved tissue penetration (281, 282). Continuous theta burst stimulation and other multimodal neuromodulation approaches also promote solute clearance and improve cognitive and emotional phenotypes by enhancing CSF–ISF exchange and perivascular inflow efficiency (283–286). Similarly, 40 Hz multisensory gamma stimulation has been observed to facilitate cortical fluid exchange, accelerate amyloid clearance, and improve cognitive performance (167, 287, 288).

Given the BBB restrictions on various therapeutic molecules, delivery strategies that target or utilize the glymphatic–mLV pathway have garnered increasing attention (289). Zhao et al. achieved nonhematogenous intracerebral delivery by employing indocyanine green-loaded PLGA nanoparticles, which accumulated in draining lymph nodes after subcutaneous injection in the neck and gradually diffused into the CSF via immune cell-mediated transport (290). Similarly, a nanostructure known as the “nanoplumber” significantly improved neurological recovery in a TRI model by continuously modulating the injured microenvironment, suppressing glial cell activation, and increasing mLV drainage (291).

### 7.5 Intervention stages and translational pathways

Building on the concept that perioperative cognitive vulnerability is critically influenced by clearance efficiency in the brain, we propose a phase-oriented framework in which the evolution of glymphatic–mLV function across postoperative phases serves as a key regulatory variable. Rather than representing a uniform deficit, clearance dysfunction unfolds dynamically, and the pace and completeness of its recovery may determine the reversibility of cognitive disturbances.

During the early recovery phase (1–7 days after surgery), the levels of systemic inflammatory markers generally decrease. However, the restoration of glymphatic flow and meningeal lymphatic drainage does not necessarily parallel this resolution and may remain functionally delayed (17, 292, 293). The mislocalization of AQP4 and reactive changes in astrocytes persist in some models, with microglia remaining in a primed state that amplifies responses to residual signals (5, 41, 294, 295). Within this clearance vulnerability window, cognitive fluctuations or declines in executive function may reflect suboptimal clearance efficiency rather than persistent inflammatory input. If the function of the clearance–reflux axis gradually recovers, cognitive abnormalities often prove reversible; however, if recovery is limited, an inflammation–clearance imbalance may form a self-reinforcing loop.

During the subacute phase (1–12 weeks after surgery), incomplete restoration of clearance function may progressively accompany structural remodeling. Sustained impairment of glymphatic transport and restricted PVS dynamics may perpetuate inefficient brain clearance, whereas reduced contractility and delayed drainage of mLVs can limit the export of brain-derived metabolites to peripheral lymphatic structures (25, 49, 85, 296). In parallel, persistent meningeal immune cell retention and a decreased inflammatory activation threshold have been reported in some models, potentially prolonging central inflammatory signaling beyond the initial peripheral trigger (130, 274, 297). During this phase, if the clearance system is not rebuilt through factors such as sleep structure restoration and improved neurovascular coupling, the reversibility of cognitive abnormalities may progressively decrease.

By the chronic phase (≥3 months after surgery), abnormalities in glymphatic–mLV function tend to persist, leading to the sustained accumulation of metabolic and inflammatory products within the brain interstitium and meningeal compartments. Animal studies suggest that the prolonged deposition of Aβ, tau, fibrinogen, and extracellular vesicles can disrupt neural network synchrony and display molecular characteristics that partially resemble those of neurodegenerative processes (13, 227, 298, 299). At this stage, PNDs may extend beyond a transient perioperative phenomenon and contribute to long-term vulnerability to cognitive decline. Accordingly, interventions should shift from targeting acute inflammation toward preserving network integrity and mitigating longer-term cognitive risk.

**Figure 7:**
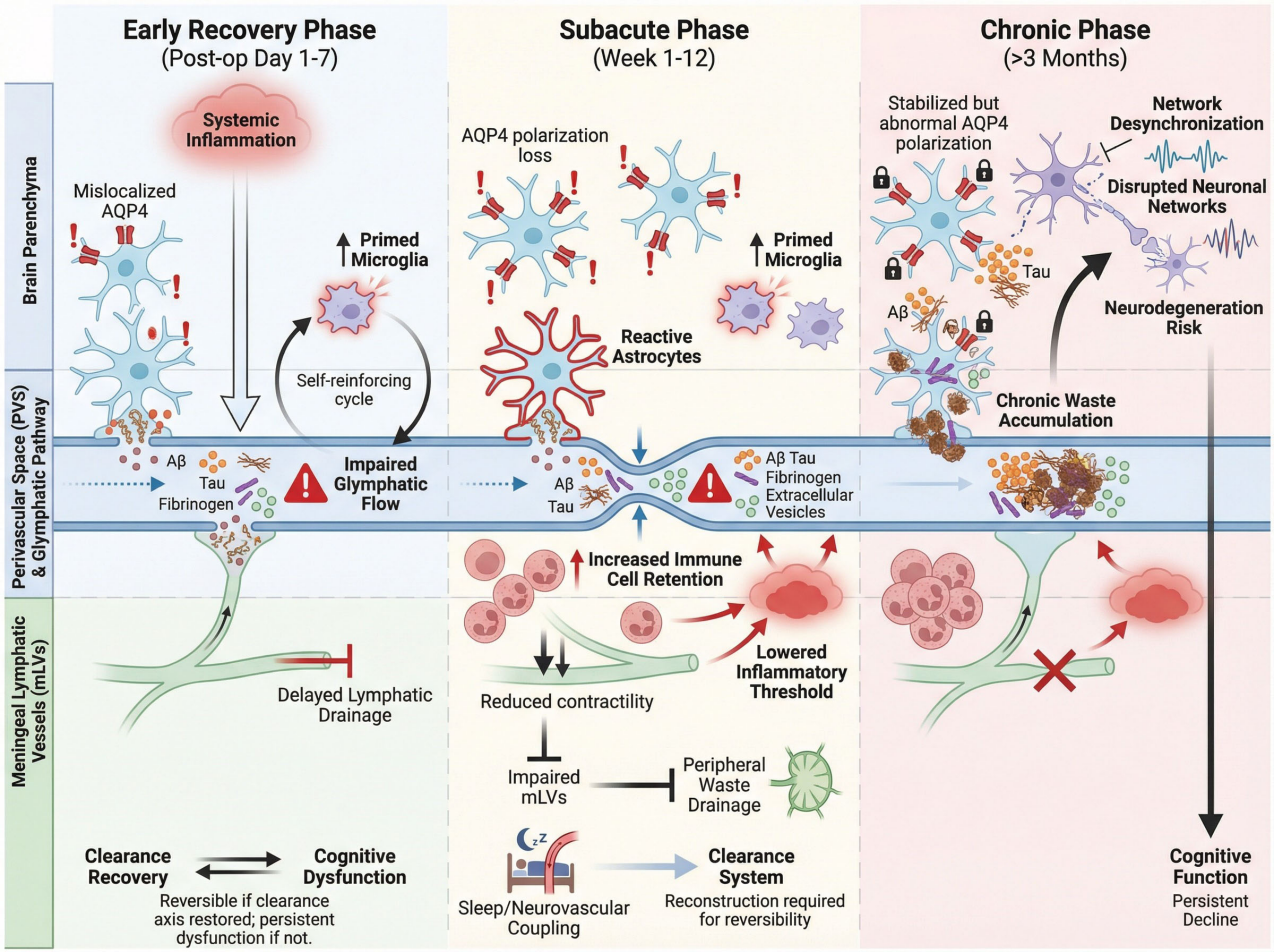
Temporal evolution of glymphatic–mLV system dysfunction and PND progression. Early inflammatory resolution amid delayed clearance creates a window of vulnerability. Subacute AQP4 mispolarization and mLV dysfunction amplify inflammation and clearance deficits, whereas chronic metabolic waste accumulation and network desynchronization drive persistent cognitive decline. The recovery speed and clearance efficiency may influence the reversibility and long-term trajectory of PNDs.

This temporal framework is not a clinical staging system but a mechanistically informed construct based on experimental evidence. It posits that the trajectory of postoperative recovery of the clearance function of the brain critically regulates the reversibility of cognitive changes. Accordingly, interventions should prioritize the restoration of glymphatic–mLV function and cerebral homeostasis. By enhancing intrinsic clearance pathways rather than solely suppressing peripheral inflammation, this approach represents a mechanism-based and potentially translatable strategy to prevent and mitigate PNDs (**Figure 7**).

### 7.6 Clinical evidence and evaluation of interventions targeting the glymphatic–mLV system in PNDs

Although several interventions targeting the glymphatic–mLV system have demonstrated potential for improving PNDs, the current clinical evidence remains limited and is largely based on indirect associations, small-sample studies, or single-center observational trials. To improve clinical interpretability, the available human studies were categorized as direct or indirect evidence according to whether they addressed the full or partial link among intervention, glymphatic–mLV modulation, and PND-related outcomes. The level of evidence (LOE) for each study was then assigned according to a modified application of the 2011 Oxford Centre for Evidence-Based Medicine Levels of Evidence (300). As summarized in **Table 3**, representative interventions—including Dex, sleep-related strategies, and neuromodulation—demonstrate promise in reducing perioperative neurocognitive risks or improving glymphatic–mLV-related function. However, the current literature is characterized not only by limited sample sizes and methodological heterogeneity but also by fragmentation of the evidence chain, as most studies support only part of the proposed pathway (rather than directly demonstrating intervention-induced glymphatic–mLV modulation in conjunction with improved PND outcomes). Therefore, the clinical applicability and mechanistic specificity of these interventions still require further validation.

**Table 3:** Evidence-based appraisal of clinical evidence relevant to glymphatic–mLV-targeted interventions in PNDs.

Evidence type	Intervention category	Specific intervention	Main findings	LOE	Major limitations	Reference
Mechanistically supported clinical evidence (Intervention → PND, with parallel evidence for glymphatic–mLV involvement)	Neuromodulation	TMS	Postoperative iTBS improved selected memory performance at postoperative day 7 and AMTS at day 30; glymphatic involvement was supported by parallel perioperative mouse data	2	Small sample; exploratory single-center trial; short follow-up	(137)
Indirect A (Intervention → PND)	Pharmacological	Dexmedetomidine	Reduced PND incidence and improved early postoperative cognitive function; long-term cognitive effects remain inconsistent	1	Heterogeneity in dosing, timing, and populations	(303–306)
	Sleep-related	Music and aromatherapy interventions	Nonpharmacological sleep interventions improve perioperative sleep and may reduce PND incidence	2	Small RCTs; heterogeneous protocols; short follow-up	(307, 308)
		Melatonin	Systematic review shows reduction of PND incidence	1	Heterogeneity in dosing and outcome measures	(309)
		Standardized sleep protocols	Meta-analysis shows sleep interventions reduce PND incidence	1	Intervention heterogeneity; variable endpoints	(310)
Indirect B (Intervention → Glymphatic–mLV)	Sleep-related	CBT-I	Improved sleep and associated with enhanced glymphatic–mLV clearance inferred from reductions in neurodegeneration biomarkers	4	Small, single-arm; exploratory; cannot infer causality	(311)
	Pharmacological	Vortioxetine	Increased glymphatic activity and improved cognition in MDD patients	3	Small sample; open-label; short follow-up	(312)
	Neuromodulation	Cerebellar iTBS	Improved glymphatic clearance and cognitive function in AD patients	2	Very small sample; single-center; short follow-up; mechanisms not fully established	(313)
		rTMS	Improved upper limb motor function and associated changes in DTI-ALPS, suggesting modulation of glymphatic–mLV activity	2	Moderate sample; single-center; DTI-ALPS as indirect proxy	(314)
	Device/Procedure	CPAP	Improved glymphatic drainage in OSA patients	3	Small sample; non-randomized design; indirect outcome	(315)
		FUS-BBB opening	Enhanced perivenous fluid transport, suggesting improved glymphatic clearance	4	Very small sample; single-arm exploratory; indirect imaging outcome	(99)
Indirect C (Glymphatic–mLV → PND)	Neuroimaging	DTI-ALPS imaging assessment	DTI-ALPS index decreased in OSA patients and correlated with early postoperative cognitive decline	3	Small, cross-sectional; cannot infer causality	(41)

## 8 Conclusions and perspectives

The integrated framework linking peripheral inflammation, an imbalance in the clearance function of the brain, and cognitive impairment offers a conceptual perspective on the pathogenesis of PNDs. Its translational relevance, however, depends on the further clarification of several fundamental questions.

The currently available evidence indicates a temporal association among inflammatory responses, altered clearance kinetics, and cognitive outcomes. However, the precise sequence and causal relationships among these processes remain unclear. Most studies rely on animal models or cross-sectional observations, and longitudinal designs that concurrently track inflammation, clearance dynamics, and cognitive trajectories within the same cohort are limited. Future studies may move beyond documenting associations to examining whether distinct periods of increased clearance vulnerability can be identified and to clarifying which regulatory nodes influence cognitive outcomes across temporal stages. Clarifying whether alterations in clearance function confer independent predictive value, rather than simply mirroring the intensity of inflammation, will be essential for assessing the mechanistic relevance of this framework.

A quantitative assessment of the clearance function of the brain remains a central translational barrier, given the limited use of perioperative tools for capturing dynamic glymphatic–mLV changes. Multimodal approaches integrating imaging features, biofluid biomarkers, and sleep–neurovascular measures over time may improve resolution.

The feasibility and safety of modulating clearance remain uncertain, and the currently available evidence does not establish a consistent clinical benefit. Any intervention must be evaluated within the broader context of systemic physiology, including infection risk, coagulation balance, and hemodynamic stability. Defining normative clearance parameters and interindividual variability may help establish rational intervention thresholds.

Mechanism-informed stratification and precise trial design may improve efficiency and interpretability. The clinical and biological heterogeneity of PNDs suggests that multiple interacting pathways contribute to the variability in outcomes. Stratification grounded in quantifiable domains, such as the inflammatory burden, vascular–coagulation status, neurodegenerative vulnerability, or clearance-related alterations, may increase signal detection and reduce the dilution effects associated with population heterogeneity (293, 301).

Long-term outcomes remain a major gap in the current evidence, as most studies focus on short-term delirium or transient cognitive changes. Well-designed prospective cohorts with extended follow-up are needed to determine whether perioperative exposure is associated with sustained cognitive trajectories and neurodegenerative processes. Clarifying this relationship would strengthen the clinical and public health relevance of perioperative brain protection strategies.

Coordinated interdisciplinary collaboration is essential for addressing these challenges. Integrating expertise from anesthesiology, neuroimmunology, geriatrics, systems biology, and data science together with standardized data sharing within multicenter networks may increase reproducibility and external validity (299). The development of predictive models grounded in mechanistic variables, rather than relying solely on traditional clinical risk scores, could facilitate a transition from passive risk identification to more proactive and monitorable perioperative brain protection strategies.

**Figure 8:**
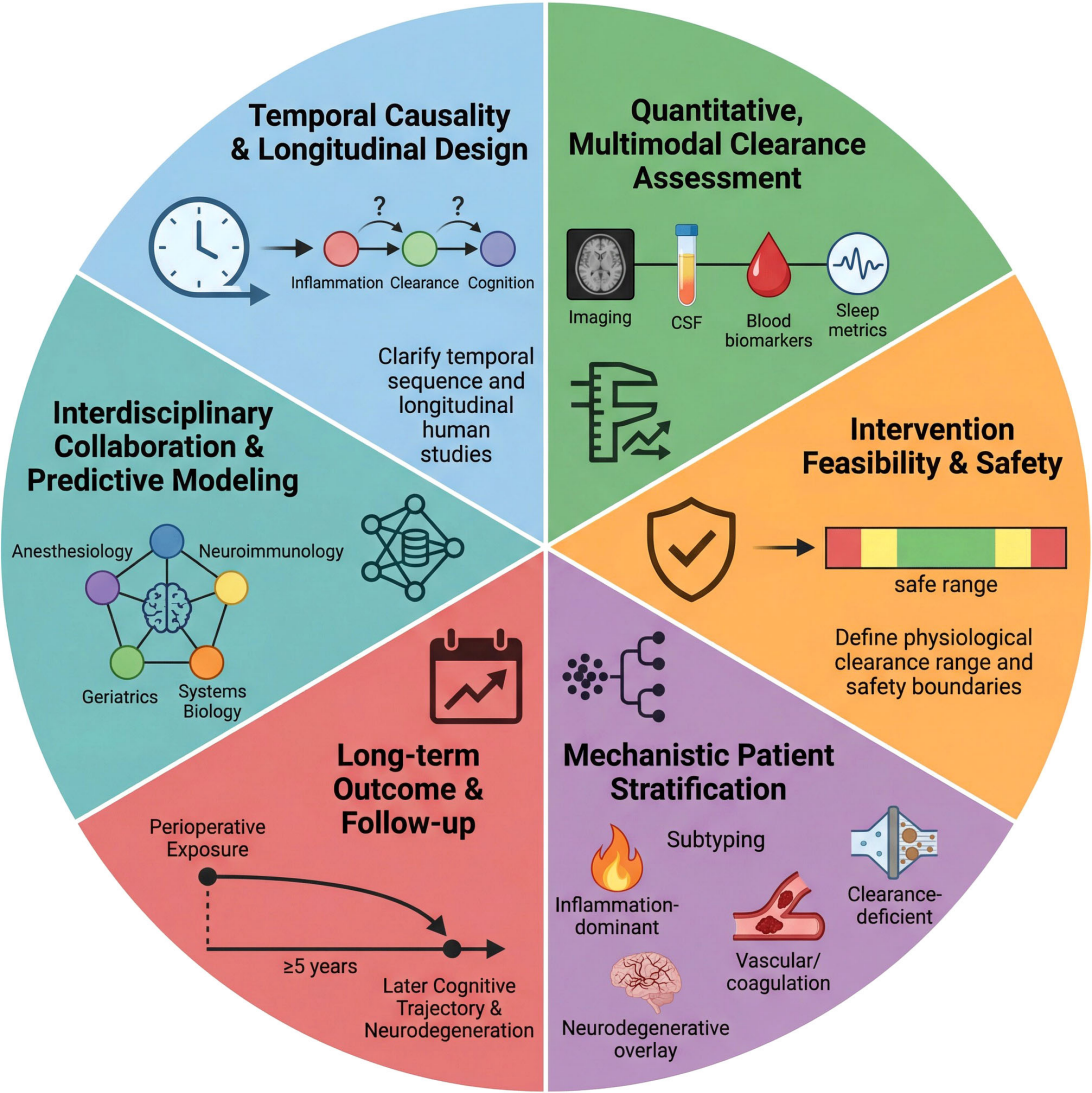
Schematic diagram of future research directions and the clinical translation framework. This diagram illustrates a potential clinical translation pathway for studying perioperative inflammation, glymphatic–mLV dysfunction, and PND outcomes. Key components include multiple time-point longitudinal designs, quantitative multimodal assessments of central clearance, patient stratification, evaluations of the feasibility and safety of interventions, and follow-up periods extending at least five years to capture cognitive and neurodegenerative outcomes. The integration of interdisciplinary collaboration and predictive modeling is also highlighted. Together, these approaches aim to inform personalized risk assessments and guide precise interventions.

A glymphatic–mLV imbalance may serve as a critical peripheral-to-central interface that links systemic inflammation, impaired brain clearance, and cognitive dysfunction in PNDs. This framework emphasizes an integrative, mechanism-based perspective rather than a single therapeutic target, highlighting testable, quantifiable processes and the adaptability of the brain to inflammatory and metabolic stress. By synthesizing the evidence on perioperative inflammation, clearance dynamics, and cognitive outcomes, it clarifies knowledge gaps, identifies research opportunities, and provides a structured foundation for translational studies aimed at defining clinically meaningful endpoints. Ultimately, this integrative model supports future efforts to preserve brain homeostasis and guide individualized perioperative risk assessments (**Figure 8**).

## Glossary

AAV: Adeno-associated virus
AD: Alzheimer disease
ALPS: Analysis along the perivascular space
AMTS: Abbreviated Mental Test Score
APOE: Apolipoprotein E
AQP4: Aquaporin-4
AV-001: Angiopoietin-1–derived peptide
Aβ: Amyloid-beta
BAMs: Border-associated macrophages
BBB: Blood–brain barrier
BCSFB: Blood–cerebrospinal fluid barrier
BDNF: Brain-derived neurotrophic factor
BMAL1: Brain and muscle ARNT-like 1
CBT-I: Cognitive Behavioral Therapy for Insomnia
CCL2: C–C motif chemokine ligand 2
CCR2: C–C chemokine receptor type 2
CNS: Central nervous system
CP: Choroid plexus
CPAP: Continuous Positive Airway Pressure
CRCI: Cancer-related cognitive impairment
CRP: C-reactive protein
CSF: Cerebrospinal fluid
CVOs: Circumventricular organs
CyTOF: Cytometry by time-of-flight (mass cytometry)
DCE-MRI: Dynamic contrast-enhanced magnetic resonance imaging
dcLNs: Deep cervical lymph nodes
DCs: Dendritic cells
Dex: Dexmedetomidine
dNCR: Delayed neurocognitive recovery
DTI: Diffusion tensor imaging
DTI-ALPS: Diffusion tensor imaging along the perivascular space
EEG: Electroencephalography
ENS: Enteric nervous system
FLAIR: Fluid-attenuated inversion recovery
FUS-BBB: Focused Ultrasound Blood-Brain Barrier opening
GFAP: Glial fibrillary acidic protein
HMGB1: High mobility group box 1
ICAM-1: Intercellular adhesion molecule-1
IL-1β: Interleukin-1β
IL-6: Interleukin-6
IPAD: Intramural periarterial drainage
ISF: Interstitial fluid
iTBS: Intermittent theta burst stimulation
KX: Ketamine–xylazine
LOE: Level of evidence
LPS: Lipopolysaccharide
LSFM: Light-sheet fluorescence microscopy
LYVE-1: Lymphatic vessel endothelial hyaluronan receptor 1
MDD: Major depressive disorder
MEG: Magnetoencephalography
MERFISH: Multiplexed error-robust fluorescence *in situ* hybridization
mLVs: Meningeal lymphatic vessels
MRI: Magnetic resonance imaging
MS: Multiple sclerosis
NCDs: Neurocognitive disorders
NfL: Neurofilament light chain
NF-κB: Nuclear factor kappa B
NIRS: Near-infrared spectroscopy
NLRP3: NLR family pyrin domain containing 3
NVU: Neurovascular unit
OSA: Obstructive sleep apnea
PAMPs: Pathogen-associated molecular patterns
p-tau: Phosphorylated tau
PC-MRI: Phase-contrast magnetic resonance imaging
PD: Parkinson disease
PET: Positron emission tomography
PNDs: Perioperative neurocognitive disorders
POCD: Postoperative cognitive dysfunction
POD: Postoperative delirium
PVS: Perivascular space
RAGE: Receptor for advanced glycation end products
RCTs: Randomized controlled trials
rTMS: Repetitive transcranial magnetic stimulation
S100β: S100 calcium-binding protein β
scRNA-seq: Single-cell RNA sequencing
snRNA-seq: Single-nucleus RNA sequencing
SPECT: Single-photon emission computed tomography
sTREM2: Soluble triggering receptor expressed on myeloid cells 2
TBI: Traumatic brain injury
Teffs: Effector T cells
TMS: Transcranial magnetic stimulation
TNF-α: Tumor necrosis factor-α
Tregs: Regulatory T cells
VCAM-1: Vascular cell adhesion molecule-1
VEGF-C: Vascular endothelial growth factor C
VEGFR3: Vascular endothelial growth factor receptor 3.

## References

[1] Ju LS Morey TE Seubert CN Martynyuk AE . Intergenerational perioperative neurocognitive disorder. Biol Bsl. (2023) 12:567. doi: 10.3390/biology12040567. PMID: 37106766 PMC10135810

[2] Safavynia SA Goldstein PA Evered LA . Mitigation of perioperative neurocognitive disorders: a holistic approach. Front Aging Neurosci. (2022) 14:949148. doi: 10.3389/fnagi.2022.949148. PMID: 35966792 PMC9363758

[3] Evered L Silbert B Knopman DS Scott DA DeKosky ST Rasmussen LS . Recommendations for the nomenclature of cognitive change associated with anaesthesia and surgery—2018. Br J Anaesth. (2018) 121:1005–12. doi: 10.1016/j.bja.2017.11.087. PMID: 30336844 PMC7069032

[4] Kong H Xu LM Wang DX . Perioperative neurocognitive disorders: a narrative review focusing on diagnosis, prevention, and treatment. CNS Neurosci Ther. (2022) 28:1147–67. doi: 10.1111/cns.13873. PMID: 35652170 PMC9253756

[5] Jia S Yang H Huang F Fan W . Systemic inflammation, neuroinflammation and perioperative neurocognitive disorders. Inflammation Res. (2023) 72:1895–907. doi: 10.1007/s00011-023-01792-2. PMID: 37688642

[6] Pereira JVB Aung Thein MZ Nitchingham A Caplan GA . Delirium in older adults is associated with development of new dementia: a systematic review and meta-analysis. Int J Geriatr Psychiatry. (2021) 36:993–1003. doi: 10.1002/gps.5508. PMID: 33638566

[7] Deiner S Liu X Lin HM Jacoby R Kim J Baxter MG . Does postoperative cognitive decline result in new disability after surgery? Ann Surg. (2021) 274:e1108–14. doi: 10.1097/SLA.0000000000003764. PMID: 32149824

[8] Jevtovic-Todorovic V Hartman RE Izumi Y Benshoff ND Dikranian K Zorumski CF . Early exposure to common anesthetic agents causes widespread neurodegeneration in the developing rat brain and persistent learning deficits. J Neurosci. (2003) 23:876–82. doi: 10.1523/JNEUROSCI.23-03-00876.2003. PMID: 12574416 PMC6741934

[9] Rappaport BA Suresh S Hertz S Evers AS Orser BA . Anesthetic neurotoxicity--clinical implications of animal models. N Engl J Med. (2015) 372:796–7. doi: 10.1056/NEJMp1414786. PMID: 25714157

[10] Evered LA Silbert BS . Postoperative cognitive dysfunction and noncardiac surgery. Anesth Analg. (2018) 127:496–505. doi: 10.1213/ANE.0000000000003514. PMID: 29889707

[11] Zhang S Liu C Sun J Li Y Lu J Xiong X . Bridging the gap: investigating the link between inflammasomes and postoperative cognitive dysfunction. Aging Dis. (2023) 14:1981–2002. doi: 10.14336/AD.2023.0501. PMID: 37450925 PMC10676784

[12] Qiu Y Mo C Xu S Chen L Ye W Kang Y . Research progress on perioperative blood-brain barrier damage and its potential mechanism. Front Cell Dev Biol. (2023) 11:1174043. doi: 10.3389/fcell.2023.1174043. PMID: 37101615 PMC10124715

[13] Zhang Q Niu Y Li Y Xia C Chen Z Chen Y . Meningeal lymphatic drainage: novel insights into central nervous system disease. Signal Transd Tgt Ther. (2025) 10:142. doi: 10.1038/s41392-025-02177-z. PMID: 40320416 PMC12050339

[14] Iliff JJ Wang M Liao Y Plogg BA Peng W Gundersen GA . A paravascular pathway facilitates CSF flow through the brain parenchyma and the clearance of interstitial solutes, including amyloid β. Sci Transl Med. (2012) 4:147ra111. doi: 10.1126/scitranslmed.3003748. PMID: 22896675 PMC3551275

[15] Proulx ST . Cerebrospinal fluid outflow: a review of the historical and contemporary evidence for arachnoid villi, perineural routes, and dural lymphatics. Cell Mol Life Sci. (2021) 78:2429–57. doi: 10.1007/s00018-020-03706-5. PMID: 33427948 PMC8004496

[16] Louveau A Smirnov I Keyes TJ Eccles JD Rouhani SJ Peske JD . Structural and functional features of central nervous system lymphatic vessels. Nature. (2015) 523:337–41. doi: 10.1038/nature14432. PMID: 26030524 PMC4506234

[17] Aspelund A Antila S Proulx ST Karlsen TV Karaman S Detmar M . A dural lymphatic vascular system that drains brain interstitial fluid and macromolecules. J Exp Med. (2015) 212:991–9. doi: 10.1084/jem.20142290. PMID: 26077718 PMC4493418

[18] Jacob L De Brito Neto J Lenck S Corcy C Benbelkacem F Geraldo LH . Conserved meningeal lymphatic drainage circuits in mice and humans. J Exp Med. (2022) 219:e20220035. doi: 10.1084/jem.20220035. PMID: 35776089 PMC9253621

[19] Yoon JH Jin H Kim HJ Hong SP Yang MJ Ahn JH . Nasopharyngeal lymphatic plexus is a hub for cerebrospinal fluid drainage. Nature. (2024) 625:768–77. doi: 10.1038/s41586-023-06899-4. PMID: 38200313 PMC10808075

[20] Song J Li ZH Xue XY Meng JC Zhu WX Hu S . Neonatal stress disrupts the glymphatic system development and increases the susceptibility to Parkinson’s disease in later life. CNS Neurosci Ther. (2024) 30:e14587. doi: 10.1111/cns.14587. PMID: 38421142 PMC10851323

[21] Locke A Kanekar S . Imaging of premature infants. Clin Perinatol. (2022) 49:641–55. doi: 10.1016/j.clp.2022.06.001. PMID: 36113927

[22] Peng T Lin Y Xu X Li J Liu M Zhang C . Assessing neonatal brain glymphatic system development using diffusion tensor imaging along the perivascular space and choroid plexus volume. BMC Med Imaging. (2025) 25:126. doi: 10.1186/s12880-025-01673-6. PMID: 40247273 PMC12007372

[23] Lin S Guo M Liang Q Lin X Chen S Li Y . Evaluation of glymphatic system development in neonatal brain via diffusion analysis along the perivascular space index. Ann Neurol. (2024) 96:970–80. doi: 10.1002/ana.27047. PMID: 39096048

[24] Dong R Han Y Lv P Jiang L Wang Z Peng L . Long-term isoflurane anesthesia induces cognitive deficits via AQP4 depolarization mediated blunted glymphatic inflammatory proteins clearance. J Cereb Blood Flow Metab. (2024) 44:1450–66. doi: 10.1177/0271678X241237073. PMID: 38443763 PMC11342724

[25] Zhu B Cao A Chen C Zhou W Luo W Gui Y . MMP-9 inhibition alleviates postoperative cognitive dysfunction by improving glymphatic function via regulating AQP4 polarity. Int Immunopharmacol. (2024) 126:111215. doi: 10.1016/j.intimp.2023.111215. PMID: 38000234

[26] Wang S Yu X Cheng L Ren W Wen G Wu X . Dexmedetomidine improves the circulatory dysfunction of the glymphatic system induced by sevoflurane through the PI3K/AKT/ΔFosB/AQP4 pathway in young mice. Cell Death Dis. (2024) 15:448. doi: 10.1038/s41419-024-06845-w. PMID: 38918408 PMC11199640

[27] Shen X Dong Y Xu Z Wang H Miao C Soriano SG . Selective anesthesia-induced neuroinflammation in developing mouse brain and cognitive impairment. Anesthesiology. (2013) 118:502–15. doi: 10.1097/ALN.0b013e3182834d77. PMID: 23314110 PMC3580002

[28] Millar K Bowman AW Burns D McLaughlin P Moores T Morton NS . Children’s cognitive recovery after day-case general anesthesia: a randomized trial of propofol or isoflurane for dental procedures. Paediatr Anaesth. (2014) 24:201–7. doi: 10.1111/pan.12316. PMID: 24330482

[29] Aun CST McBride C Lee A Lau ASC Chung RCK Yeung CK . Short-term changes in postoperative cognitive function in children aged 5 to 12 years undergoing general anesthesia: a cohort study. Med Baltimore. (2016) 95:e3250. doi: 10.1097/MD.0000000000003250. PMID: 27057869 PMC4998785

[30] Han F Wang X Zhang H Wang J Bao Z Li Y . Predictors and occurrence of postoperative cognitive dysfunction in children undergoing noncardiac surgery: a prospective cohort study. Ibrain. (2023) 9:148–56. doi: 10.1002/ibra.12066. PMID: 37786547 PMC10528770

[31] Xiong Y Yu Q Zhi H Peng H Xie M Li R . Advances in the study of the glymphatic system and aging. CNS Neurosci Ther. (2024) 30:e14803. doi: 10.1111/cns.14803. PMID: 38887168 PMC11183173

[32] Achariyar TM Li B Peng W Verghese PB Shi Y McConnell E . Glymphatic distribution of CSF-derived apoE into brain is isoform specific and suppressed during sleep deprivation. Mol Neurodegener. (2016) 11:74. doi: 10.1186/s13024-016-0138-8. PMID: 27931262 PMC5146863

[33] Kuonqui K Campbell AC Sarker A Roberts A Pollack BL Park HJ . Dysregulation of lymphatic endothelial VEGFR3 signaling in disease. Cells. (2023) 13:68. doi: 10.3390/cells13010068. PMID: 38201272 PMC10778007

[34] Liukkonen M Heloterä H Siintamo L Ghimire B Mattila P Kivinen N . Oxidative stress and inflammation-related mRNAs are elevated in serum of a Finnish wet AMD cohort. Invest Ophthalmol Vis Sci. (2024) 65:30. doi: 10.1167/iovs.65.13.30. PMID: 39546296 PMC11578155

[35] Hsu SJ Zhang C Jeong J Lee SI McConnell M Utsumi T . Enhanced meningeal lymphatic drainage ameliorates neuroinflammation and hepatic encephalopathy in cirrhotic rats. Gastroenterology. (2021) 160:1315–1329.e13. doi: 10.1053/j.gastro.2020.11.036. PMID: 33227282 PMC7956141

[36] Rizo-Téllez SA Sekheri M Filep JG . C-reactive protein: a target for therapy to reduce inflammation. Front Immunol. (2023) 14:1237729. doi: 10.3389/fimmu.2023.1237729. PMID: 37564640 PMC10410079

[37] Bremer AS Henschel N Burkard H Bernis ME Ulas T Sabir H . Transcriptomic profile of microglia following inflammation-sensitized hypoxic-ischemic brain injury in neonatal rats suggests strong contribution to neutrophil chemotaxis and activation. J Neuroinflamm. (2025) 22:189. doi: 10.1186/s12974-025-03516-1. PMID: 40684206 PMC12276676

[38] Heinrich M Sieg M Kruppa J Nürnberg P Schreier PH Heilmann-Heimbach S . Association between genetic variants of the cholinergic system and postoperative delirium and cognitive dysfunction in elderly patients. BMC Med Genomics. (2021) 14:248. doi: 10.1186/s12920-021-01071-1. PMID: 34674705 PMC8529799

[39] Travica N Aslam H O’Neil A Lane MM Berk M Gamage E . Brain derived neurotrophic factor in perioperative neurocognitive disorders: current evidence and future directions. Neurobiol Learn Mem. (2022) 193:107656. doi: 10.1016/j.nlm.2022.107656. PMID: 35792324

[40] Connal S . Perioperative neurocognitive disorders. Br J Hosp Med Lond. (2023) 84:1–2. doi: 10.12968/hmed.2023.0184. PMID: 37646546

[41] Roy B Kumar R Sarovich SD Vacas S . The role of the glymphatic system in perioperative neurocognitive disorders. J Neurosurg Anesthesiol. (2025) 37:181–7. doi: 10.1097/ANA.0000000000000973. PMID: 38775193 PMC11582080

[42] Ye L Cheng X Shi Y Liu Z Xiong Y Huang Y . Long non-coding RNA MEG3 alleviates postoperative cognitive dysfunction by suppressing inflammatory response and oxidative stress via has-mir-106a-5p/SIRT3. Neuroreport. (2023) 34:357–67. doi: 10.1097/WNR.0000000000001901. PMID: 36966803

[43] Walters JL Chelonis JJ Fogle CM Orser BA Paule MG . Single and repeated exposures to the volatile anesthetic isoflurane do not impair operant performance in aged rats. Neurotoxicology. (2016) 56:159–69. doi: 10.1016/j.neuro.2016.07.012. PMID: 27498192

[44] Han G Zhou Y Zhang K Jiao B Hu J Zhang Y . Age- and time-of-day dependence of glymphatic function in the human brain measured via two diffusion MRI methods. Front Aging Neurosci. (2023) 15:1173221. doi: 10.3389/fnagi.2023.1173221. PMID: 37284019 PMC10239807

[45] Zhou Y Cai J Zhang W Gong X Yan S Zhang K . Impairment of the glymphatic pathway and putative meningeal lymphatic vessels in the aging human. Ann Neurol. (2020) 87:357–69. doi: 10.1002/ana.25670. PMID: 31916277

[46] Ahn JH Cho H Kim JH Kim SH Ham JS Park I . Meningeal lymphatic vessels at the skull base drain cerebrospinal fluid. Nature. (2019) 572:62–6. doi: 10.1038/s41586-019-1419-5. PMID: 31341278

[47] Li G Cao Y Tang X Huang J Cai L Zhou L . The meningeal lymphatic vessels and the glymphatic system: potential therapeutic targets in neurological disorders. J Cereb Blood Flow Metab. (2022) 42:1364–82. doi: 10.1177/0271678X221098145. PMID: 35484910 PMC9274866

[48] Guo X Zhang G Peng Q Huang L Zhang Z Zhang Z . Emerging roles of meningeal lymphatic vessels in Alzheimer’s disease. J Alzheimers Dis. (2023) 94:S355–66. doi: 10.3233/JAD-221016. PMID: 36683509 PMC10473149

[49] Gao X Ming J Liu S Lai B Fang F Cang J . Sevoflurane enhanced the clearance of Aβ1–40 in hippocampus under surgery via up-regulating AQP-4 expression in astrocyte. Life Sci. (2019) 221:143–51. doi: 10.1016/j.lfs.2019.02.024. PMID: 30763576

[50] Wu L Liu Y He Q Ao G Xu N He W . PEDF-34 attenuates neurological deficit and suppresses astrocyte-dependent neuroinflammation by modulating astrocyte polarization via 67LR/JNK/STAT1 signaling pathway after subarachnoid hemorrhage in rats. J Neuroinflamm. (2024) 21:178. doi: 10.1186/s12974-024-03171-y. PMID: 39034417 PMC11264993

[51] Mäkinen T . Lymphatic vessels at the base of the mouse brain provide direct drainage to the periphery. Nature. (2019) 572:34–5. doi: 10.1038/d41586-019-02166-7. PMID: 31358927

[52] Ang PS Zhang DM Azizi SA Norton de Matos SA Brorson JR . The glymphatic system and cerebral small vessel disease. J Stroke Cerebrovasc Dis. (2024) 33:107557. doi: 10.1016/j.jstrokecerebrovasdis.2024.107557. PMID: 38198946 PMC11579894

[53] Tian B Zhao C Liang JL Zhang HT Xu YF Zheng HL . Glymphatic function and its influencing factors in different glucose metabolism states. World J Diabetes. (2024) 15:1537–50. doi: 10.4239/wjd.v15.i7.1537. PMID: 39099805 PMC11292332

[54] Yang G Deng N Liu Y Gu Y Yao X . Evaluation of glymphatic system using diffusion MR technique in T2DM cases. Front Hum Neurosci. (2020) 14:300. doi: 10.3389/fnhum.2020.00300. PMID: 32922272 PMC7456821

[55] Song K Zhang R Zhao X Yang L Wang Q Gao W . Perioperative neurocognitive disorder changes in elderly diabetes patients within 30 days after surgery: a retrospective cohort study. Aging Clin Exp Res. (2023) 35:2911–8. doi: 10.1007/s40520-023-02583-9. PMID: 37847351

[56] Wang L Chen B Liu T Luo T Kang W Liu W . Risk factors for delayed neurocognitive recovery in elderly patients undergoing thoracic surgery. BMC Anesthesiol. (2023) 23:102. doi: 10.1186/s12871-023-02056-6. PMID: 37003967 PMC10064736

[57] Ntalouka MP Arnaoutoglou E Vrakas S Staikou C Angelis FA Papadopoulos G . The effect of type 2 diabetes mellitus on perioperative neurocognitive disorders in patients undergoing elective noncardiac surgery under general anesthesia a prospective cohort study. J Anaesthesiol Clin Pharmacol. (2022) 38:252–62. doi: 10.4103/joacp.JOACP_292_20. PMID: 36171952 PMC9511857

[58] Abdul Y Li W Ward R Abdelsaid M Hafez S Dong G . Deferoxamine treatment prevents post-stroke vasoregression and neurovascular unit remodeling leading to improved functional outcomes in type 2 male diabetic rats: role of endothelial ferroptosis. Transl Stroke Res. (2021) 12:615–30. doi: 10.1007/s12975-020-00844-7. PMID: 32875455 PMC7917163

[59] Mortensen KN Sanggaard S Mestre H Lee H Kostrikov S Xavier ALR . Impaired glymphatic transport in spontaneously hypertensive rats. J Neurosci. (2019) 39:6365–77. doi: 10.1523/JNEUROSCI.1974-18.2019. PMID: 31209176 PMC6687896

[60] Xia Y Lyu C Chen P Jiang Y Qu C Lyu X . The glymphatic system was impaired in spontaneously hypertensive rats. Sci Rep. (2025) 15:18321. doi: 10.1038/s41598-025-02054-3. PMID: 40419570 PMC12106702

[61] Li YL Huang HF Le Y . Risk factors and predictive value of perioperative neurocognitive disorders in elderly patients with gastrointestinal tumors. BMC Anesthesiol. (2021) 21:193. doi: 10.1186/s12871-021-01405-7. PMID: 34281529 PMC8287702

[62] Huang SY Zhang YR Guo Y Du J Ren P Wu BS . Glymphatic system dysfunction predicts amyloid deposition, neurodegeneration, and clinical progression in Alzheimer’s disease. Alzheimers Dement. (2024) 20:3251–69. doi: 10.1002/alz.13789. PMID: 38501315 PMC11095446

[63] Li Y Li YJ Fang X Chen DQ Yu WQ Zhu ZQ . Peripheral inflammation as a potential mechanism and preventive strategy for perioperative neurocognitive disorder under general anesthesia and surgery. Front Cell Neurosci. (2024) 18:1365448. doi: 10.3389/fncel.2024.1365448. PMID: 39022312 PMC11252726

[64] Giannetto M Xia M Stæger FF Metcalfe T Vinitsky HS Dang JAML . Biological sex does not predict glymphatic influx in healthy young, middle aged or old mice. Sci Rep. (2020) 10:16073. doi: 10.1038/s41598-020-72621-3. PMID: 32999319 PMC7528110

[65] Zhang Y Zhang R Ye Y Wang S Jiaerken Y Hong H . The influence of demographics and vascular risk factors on glymphatic function measured by diffusion along perivascular space. Front Aging Neurosci. (2021) 13:693787. doi: 10.3389/fnagi.2021.693787. PMID: 34349635 PMC8328397

[66] Wang H Wang B Normoyle KP Jackson K Spitler K Sharrock MF . Brain temperature and its fundamental properties: a review for clinical neuroscientists. Front Neurosci. (2014) 8:307. doi: 10.3389/fnins.2014.00307. PMID: 25339859 PMC4189373

[67] Gu W Bai Y Cai J Mi H Bao Y Zhao X . Hypothermia impairs glymphatic drainage in traumatic brain injury as assessed by dynamic contrast-enhanced MRI with intrathecal contrast. Front Neurosci. (2023) 17:1061039. doi: 10.3389/fnins.2023.1061039. PMID: 36816105 PMC9932501

[68] Duan Y Wu D Huber M Shi J An H Wei W . New endovascular approach for hypothermia with intrajugular cooling and neuroprotective effect in ischemic stroke. Stroke. (2020) 51:628–36. doi: 10.1161/STROKEAHA.119.026523. PMID: 31884905

[69] Que M Li S Xia Q Li X Luo X Zhan G . Microbiota-gut-brain axis in perioperative neurocognitive and depressive disorders: pathogenesis to treatment. Neurobiol Dis. (2024) 200:106627. doi: 10.1016/j.nbd.2024.106627. PMID: 39111702

[70] Sun Y Wang K Zhao W . Gut microbiota in perioperative neurocognitive disorders: current evidence and future directions. Front Immunol. (2023) 14:1178691. doi: 10.3389/fimmu.2023.1178691. PMID: 37215136 PMC10192759

[71] Shukla PK Fatma S Khan MM . Editorial: gut dysbiosis-induced systemic inflammation in neurological diseases and disorders. Front Immunol. (2024) 15:1437651. doi: 10.3389/fimmu.2024.1437651. PMID: 38903516 PMC11188375

[72] He XF Li LL Xian WB Li MY Zhang LY Xu JH . Chronic colitis exacerbates NLRP3-dependent neuroinflammation and cognitive impairment in middle-aged brain. J Neuroinflamm. (2021) 18:153. doi: 10.1186/s12974-021-02199-8. PMID: 34229722 PMC8262017

[73] Koh HJ Joo J . The role of cytokines in perioperative neurocognitive disorders: a review in the context of anesthetic care. Biomedicines. (2025) 13:506. doi: 10.3390/biomedicines13020506. PMID: 40002918 PMC11853096

[74] Chen Z Maqbool J Sajid F Hussain G Sun T . Human gut microbiota and its association with pathogenesis and treatments of neurodegenerative diseases. Microb Pathog. (2021) 150:104675. doi: 10.1016/j.micpath.2020.104675. PMID: 33352217

[75] Mou Y Du Y Zhou L Yue J Hu X Liu Y . Gut microbiota interact with the brain through systemic chronic inflammation: implications on neuroinflammation, neurodegeneration, and aging. Front Immunol. (2022) 13:796288. doi: 10.3389/fimmu.2022.796288. PMID: 35464431 PMC9021448

[76] Che J Sun Y Deng Y Zhang J . Blood-brain barrier disruption: a culprit of cognitive decline? Fluid Bar CNS. (2024) 21:63. doi: 10.1186/s12987-024-00563-3. PMID: 39113115 PMC11305076

[77] Garcia-Gallardo A Campbell M . Understanding the blood-brain barrier: from physiology to pathology. Adv Exp Med Biol. (2025) 1477:1–33. doi: 10.1007/978-3-031-89525-8_1. PMID: 40442381

[78] Barisano G Lynch KM Sibilia F Lan H Shih NC Sepehrband F . Imaging perivascular space structure and function using brain MRI. Neuroimage. (2022) 257:119329. doi: 10.1016/j.neuroimage.2022.119329. PMID: 35609770 PMC9233116

[79] Yamamoto EA Bagley JH Geltzeiler M Sanusi OR Dogan A Liu JJ . The perivascular space is a conduit for cerebrospinal fluid flow in humans: a proof-of-principle report. Proc Natl Acad Sci USA. (2024) 121:e2407246121. doi: 10.1073/pnas.2407246121. PMID: 39374384 PMC11494350

[80] Rasmussen MK Mestre H Nedergaard M . Fluid transport in the brain. Physiol Rev. (2022) 102:1025–151. doi: 10.1152/physrev.00031.2020. PMID: 33949874 PMC8897154

[81] Wen C Gan JH Liu S Lu H Wang LC Wu H . Enlarged perivascular spaces correlate with blood-brain barrier leakage and cognitive impairment in Alzheimer’s disease. J Alzheimers Dis. (2025) 104:382–92. doi: 10.1177/13872877251317220. PMID: 39924914

[82] Lyu Z Chan Y Li Q Zhang Q Liu K Xiang J . Destructive effects of pyroptosis on homeostasis of neuron survival associated with the dysfunctional BBB-glymphatic system and amyloid-beta accumulation after cerebral ischemia/reperfusion in rats. Neural Plast. (2021) 2021:4504363. doi: 10.1155/2021/4504363. PMID: 34434229 PMC8382555

[83] Terrando N Monaco C Ma D Foxwell BMJ Feldmann M Maze M . Tumor necrosis factor-α triggers a cytokine cascade yielding postoperative cognitive decline. Proc Natl Acad Sci USA. (2010) 107:20518–22. doi: 10.1073/pnas.1014557107. PMID: 21041647 PMC2996666

[84] Zhang Z Jiang J He Y Cai J Xie J Wu M . Pregabalin mitigates microglial activation and neuronal injury by inhibiting HMGB1 signaling pathway in radiation-induced brain injury. J Neuroinflamm. (2022) 19:231. doi: 10.1186/s12974-022-02596-7. PMID: 36131309 PMC9490947

[85] Liu X Wu G Tang N Li L Liu C Wang F . Glymphatic drainage blocking aggravates brain edema, neuroinflammation via modulating TNF-α, IL-10, and AQP4 after intracerebral hemorrhage in rats. Front Cell Neurosci. (2021) 15:784154. doi: 10.3389/fncel.2021.784154. PMID: 34975411 PMC8718698

[86] Xin X Zhang H Yang C Wang X Zhang L Ma J . The critical role of matrix metalloproteinase 9-mediated microglial polarization in perioperative neurocognitive disorders of aged rats. Front Immunol. (2025) 16:1650254. doi: 10.3389/fimmu.2025.1650254. PMID: 40918113 PMC12408272

[87] Liu LF Hu Y Liu YN Shi DW Liu C Da X . Reactive oxygen species contribute to delirium-like behavior by activating CypA/MMP9 signaling and inducing blood-brain barrier impairment in aged mice following anesthesia and surgery. Front Aging Neurosci. (2022) 14:1021129. doi: 10.3389/fnagi.2022.1021129. PMID: 36337710 PMC9629746

[88] Hu Y Hu XD He ZQ Liu Y Gui YK Zhu SH . Anesthesia/surgery activate MMP9 leading to blood-brain barrier disruption, triggering neuroinflammation and POD-like behavior in aged mice. Int Immunopharmacol. (2024) 135:112290. doi: 10.1016/j.intimp.2024.112290. PMID: 38796964

[89] Ji Y Huang W Chen Y Zhang X Wu F Tang W . Inhibition of MMP-2 and MMP-9 attenuates surgery-induced cognitive impairment in aged mice. Brain Res Bull. (2023) 204:110810. doi: 10.1016/j.brainresbull.2023.110810. PMID: 37939860

[90] Payne T Taylor J Kunkel D Konieczka K Ingram F Blennow K . Association of preoperative to postoperative change in cerebrospinal fluid fibrinogen with postoperative delirium. BJA Open. (2024) 12:100349. doi: 10.1016/j.bjao.2024.100349. PMID: 39429436 PMC11490679

[91] Hildenborg M Kåhlin J Granath F Schening A Granström A Ebberyd A . The neuroimmune response to surgery - an exploratory study of trauma-induced changes in innate immunity and heart rate variability. Front Immunol. (2022) 13:911744. doi: 10.3389/fimmu.2022.911744. PMID: 35874666 PMC9301672

[92] Sadeghi M Peeri M Hosseini MJ . Adolescent voluntary exercise attenuated hippocampal innate immunity responses and depressive-like behaviors following maternal separation stress in male rats. Physiol Behav. (2016) 163:177–83. doi: 10.1016/j.physbeh.2016.05.017. PMID: 27184238

[93] Wang X Jiang S Cao T Huang P Di L Li J . Fibrinogen contributes to myelin deficit and cognitive impairment in aged mice after anesthesia and surgery. J Cereb Blood Flow Metab. (2026) 46:105–17. doi: 10.1177/0271678X251338953. PMID: 40770921 PMC12331660

[94] Wang X Jiang S Di L Li S Chen H Huang P . Fibrinogen drives neuroinflammation and neuropathology in perioperative neurocognitive disorders. Int Immunopharmacol. (2026) 168:115906. doi: 10.1016/j.intimp.2025.115906. PMID: 41273843

[95] Hladky SB Barrand MA . The glymphatic hypothesis: the theory and the evidence. Fluid Bar CNS. (2022) 19:9. doi: 10.1186/s12987-021-00282-z. PMID: 35115036 PMC8815211

[96] Izadi N Solár P Hašanová K Zamani A Akbar MS Mrázová K . Breaking boundaries: role of the brain barriers in metastatic process. Fluid Bar CNS. (2025) 22:3. doi: 10.1186/s12987-025-00618-z. PMID: 39780275 PMC11708195

[97] Chen T Dai Y Hu C Lin Z Wang S Yang J . Cellular and molecular mechanisms of the blood-brain barrier dysfunction in neurodegenerative diseases. Fluid Bar CNS. (2024) 21:60. doi: 10.1186/s12987-024-00557-1. PMID: 39030617 PMC11264766

[98] Verheggen ICM Van Boxtel MPJ Verhey FRJ Jansen JFA Backes WH . Interaction between blood-brain barrier and glymphatic system in solute clearance. Neurosci Biobehav Rev. (2018) 90:26–33. doi: 10.1016/j.neubiorev.2018.03.028. PMID: 29608988

[99] Mehta RI Carpenter JS Mehta RI Haut MW Wang P Ranjan M . Ultrasound-mediated blood-brain barrier opening uncovers an intracerebral perivenous fluid network in persons with Alzheimer’s disease. Fluid Bar CNS. (2023) 20:46. doi: 10.1186/s12987-023-00447-y. PMID: 37328855 PMC10276371

[100] Rowsthorn E Cribb L Sinclair B Pham W Chong TTJ Yiallourou S . Relationships between measures of neurovascular integrity and fluid transport in aging: a multi-modal neuroimaging study. Fluid Bar CNS. (2025) 22:59. doi: 10.1186/s12987-025-00664-7. PMID: 40524195 PMC12168365

[101] Hong H Tozer DJ Chen Y Brown RB Low A Markus HS . Perivascular space dysfunction in cerebral small vessel disease is related to neuroinflammation. Brain. (2025) 148:1540–50. doi: 10.1093/brain/awae357. PMID: 39509331 PMC12073995

[102] Salman MM Kitchen P Halsey A Wang MX Törnroth-Horsefield S Conner AC . Emerging roles for dynamic aquaporin-4 subcellular relocalization in CNS water homeostasis. Brain. (2022) 145:64–75. doi: 10.1093/brain/awab311. PMID: 34499128 PMC9088512

[103] Gao W Kim MW Dykstra T Du S Boskovic P Lichti CF . Engineered T cell therapy for central nervous system injury. Nature. (2024) 634:693–701. doi: 10.1038/s41586-024-07906-y. PMID: 39232158

[104] Terrabuio E Zenaro E Constantin G . The role of the CD8+ T cell compartment in ageing and neurodegenerative disorders. Front Immunol. (2023) 14:1233870. doi: 10.3389/fimmu.2023.1233870. PMID: 37575227 PMC10416633

[105] Laaker C Baenen C Kovács KG Sandor M Fabry Z . Immune cells as messengers from the CNS to the periphery: the role of the meningeal lymphatic system in immune cell migration from the CNS. Front Immunol. (2023) 14:1233908. doi: 10.3389/fimmu.2023.1233908. PMID: 37662908 PMC10471710

[106] Mukherjee U Chowdhury S Nagada KK Hasan MM Ghosh B Joshi A . Glymphatic system and intracerebral hemorrhage: identifying molecular targets for future therapeutic advancements. Ageing Res Rev. (2025) 112:102900. doi: 10.1016/j.arr.2025.102900. PMID: 40998175

[107] Cao T Jiang S Di L Huang P Cao L Wang X . Omega-3 polyunsaturated fatty acids prevent Sevoflurane-induced cognitive and fine motor dysfunctions in neonatal mice by enhancing phosphorylated tau glymphatic system clearance pathway. Mol Neurobiol. (2025) 63:228. doi: 10.1007/s12035-025-05363-w. PMID: 41324831

[108] Venkat P Culmone L Chopp M Landschoot-Ward J Wang F Zacharek A . HUCBC treatment improves cognitive outcome in rats with vascular dementia. Front Aging Neurosci. (2020) 12:258. doi: 10.3389/fnagi.2020.00258. PMID: 32973489 PMC7461871

[109] Szlufik S Kopeć K Szleszkowski S Koziorowski D . Glymphatic system pathology and neuroinflammation as two risk factors of neurodegeneration. Cells. (2024) 13:286. doi: 10.3390/cells13030286. PMID: 38334678 PMC10855155

[110] Zhao H Sun M Zhang Y Kong W Fan L Wang K . Connecting the dots: the cerebral lymphatic system as a bridge between the central nervous system and peripheral system in health and disease. Aging Dis. (2024) 15:115–52. doi: 10.14336/AD.2023.0516. PMID: 37307828 PMC10796102

[111] Yao XY Gao MC Bai SW Xie L Song YY Ding J . Enlarged perivascular spaces, neuroinflammation and neurological dysfunction in NMOSD patients. Front Immunol. (2022) 13:966781. doi: 10.3389/fimmu.2022.966781. PMID: 36248814 PMC9557144

[112] Wang YJ Sun YR Pei YH Ma HW Mu YK Qin LH . The lymphatic drainage systems in the brain: a novel target for ischemic stroke? Neural Regener Res. (2023) 18:485–91. doi: 10.4103/1673-5374.346484. PMID: 36018151 PMC9727443

[113] Bohr T Hjorth PG Holst SC Hrabětová S Kiviniemi V Lilius T . The glymphatic system: current understanding and modeling. iScience. (2022) 25:104987. doi: 10.1016/j.isci.2022.104987. PMID: 36093063 PMC9460186

[114] Iliff JJ Wang M Zeppenfeld DM Venkataraman A Plog BA Liao Y . Cerebral arterial pulsation drives paravascular CSF-interstitial fluid exchange in the murine brain. J Neurosci. (2013) 33:18190–9. doi: 10.1523/JNEUROSCI.1592-13.2013. PMID: 24227727 PMC3866416

[115] Dreha-Kulaczewski S Joseph AA Merboldt KD Ludwig HC Gärtner J Frahm J . Identification of the upward movement of human CSF *in vivo* and its relation to the brain venous system. J Neurosci. (2017) 37:2395–402. doi: 10.1523/JNEUROSCI.2754-16.2017. PMID: 28137972 PMC6596847

[116] Xie L Kang H Xu Q Chen MJ Liao Y Thiyagarajan M . Sleep drives metabolite clearance from the adult brain. Science. (2013) 342:373–7. doi: 10.1126/science.1241224. PMID: 24136970 PMC3880190

[117] Vera Quesada CL Rao SB Torp R Eide PK . Widespread distribution of lymphatic vessels in human dura mater remote from sinus veins. Front Cell Dev Biol. (2023) 11:1228344. doi: 10.3389/fcell.2023.1228344. PMID: 37795263 PMC10546208

[118] Vera Quesada CL Rao SB Torp R Eide PK . Immunohistochemical visualization of lymphatic vessels in human dura mater: methodological perspectives. Fluid Bar CNS. (2023) 20:23. doi: 10.1186/s12987-023-00426-3. PMID: 36978127 PMC10044429

[119] Shah T Leurgans SE Mehta RI Yang J Galloway CA De Mesy Bentley KL . Arachnoid granulations are lymphatic conduits that communicate with bone marrow and dura-arachnoid stroma. J Exp Med. (2023) 220:e20220618. doi: 10.1084/jem.20220618. PMID: 36469302 PMC9728136

[120] Rangroo Thrane V Thrane AS Plog BA Thiyagarajan M Iliff JJ Deane R . Paravascular microcirculation facilitates rapid lipid transport and astrocyte signaling in the brain. Sci Rep. (2013) 3:2582. doi: 10.1038/srep02582. PMID: 24002448 PMC3761080

[121] Lundgaard I Li B Xie L Kang H Sanggaard S Haswell JDR . Direct neuronal glucose uptake heralds activity-dependent increases in cerebral metabolism. Nat Commun. (2015) 6:6807. doi: 10.1038/ncomms7807. PMID: 25904018 PMC4410436

[122] Jessen NA Munk ASF Lundgaard I Nedergaard M . The glymphatic system: a beginner’s guide. Neurochem Res. (2015) 40:2583–99. doi: 10.1007/s11064-015-1581-6. PMID: 25947369 PMC4636982

[123] Iliff JJ Chen MJ Plog BA Zeppenfeld DM Soltero M Yang L . Impairment of glymphatic pathway function promotes tau pathology after traumatic brain injury. J Neurosci. (2014) 34:16180–93. doi: 10.1523/JNEUROSCI.3020-14.2014. PMID: 25471560 PMC4252540

[124] Yu Y Chen K . Peripheral immune and metabolic regulation of Aβ and tau by exercise in Alzheimer’s disease. Front Immunol. (2025) 16:1678526. doi: 10.3389/fimmu.2025.1678526. PMID: 41169370 PMC12568357

[125] He XF Liu DX Zhang Q Liang FY Dai GY Zeng JS . Voluntary exercise promotes glymphatic clearance of amyloid beta and reduces the activation of astrocytes and microglia in aged mice. Front Mol Neurosci. (2017) 10:144. doi: 10.3389/fnmol.2017.00144. PMID: 28579942 PMC5437122

[126] Giff AE Wruble Clark M Bhattacharyya S Sage PT Madore B Guenette JP . Deep cervical lymph node analysis in central nervous system inflammatory disease. Front Immunol. (2026) 17:1747114. doi: 10.3389/fimmu.2026.1747114. PMID: 41668753 PMC12883371

[127] Jin R Chan AKY Wu J Lee TMC . Relationships between inflammation and age-related neurocognitive changes. Int J Mol Sci. (2022) 23:12573. doi: 10.3390/ijms232012573. PMID: 36293430 PMC9604276

[128] Ma Q Ineichen BV Detmar M Proulx ST . Outflow of cerebrospinal fluid is predominantly through lymphatic vessels and is reduced in aged mice. Nat Commun. (2017) 8:1434. doi: 10.1038/s41467-017-01484-6. PMID: 29127332 PMC5681558

[129] Li R Zhao M Yao D Zhou X Lenahan C Wang L . The role of the astrocyte in subarachnoid hemorrhage and its therapeutic implications. Front Immunol. (2022) 13:1008795. doi: 10.3389/fimmu.2022.1008795. PMID: 36248855 PMC9556431

[130] Dikiy S Rudensky AY . Principles of regulatory T cell function. Immunity. (2023) 56:240–55. doi: 10.1016/j.immuni.2023.01.004. PMID: 36792571

[131] Zuo Y Li S Zhu X Yang J Hu X Wen W . Monocyte-derived cells promote transient insult-induced brain injury by enhancing CD8+ T cell response. Cell Rep. (2025) 44:116528. doi: 10.1016/j.celrep.2025.116528. PMID: 41205172

[132] Ji G Wang P Kong Z Cao X Shi X Feng H . Remodeling the tumor microenvironment: regulatory effects of β-sitosterol and luteolin on the immunosuppressive milieu in endometrial carcinoma and implications for combinatorial immunotherapy. Front Immunol. (2025) 16:1669606. doi: 10.3389/fimmu.2025.1669606. PMID: 41479912 PMC12753467

[133] Skinner DD Syage AR Olivarria GM Stone C Hoglin B Lane TE . Sustained infiltration of neutrophils into the CNS results in increased demyelination in a viral-induced model of multiple sclerosis. Front Immunol. (2022) 13:931388. doi: 10.3389/fimmu.2022.931388. PMID: 36248905 PMC9562915

[134] Fornari Laurindo L Aparecido Dias J Cressoni Araújo A Torres Pomini K MaChado Galhardi C Rucco Penteado Detregiachi C . Immunological dimensions of neuroinflammation and microglial activation: exploring innovative immunomodulatory approaches to mitigate neuroinflammatory progression. Front Immunol. (2023) 14:1305933. doi: 10.3389/fimmu.2023.1305933. PMID: 38259497 PMC10800801

[135] Quiroga R Sanhueza S Sepúlveda C Antilef B Muñoz C Cabrera C . CXCL9 and CXCL10 support the exacerbated humoral response in recovered COVID-19 patients who developed acute respiratory distress syndrome by promoting plasma cell differentiation, whereas CXCL9 also induces CD40L and CXCR3 upregulation on T helper cells. Front Immunol. (2025) 16:1684704. doi: 10.3389/fimmu.2025.1684704. PMID: 41425601 PMC12711852

[136] Casciano F Severi P Marongiu L Caproni A Terranova C Spitilli A . Highly differentiated T cells link systemic and vascular inflammation in a mouse model of recurrent psoriasis. Front Immunol. (2025) 16:1574455. doi: 10.3389/fimmu.2025.1574455. PMID: 40599782 PMC12208849

[137] Feng X Liu J Liang Z Du X Zhao L Xie N . Transcranial magnetic stimulation mitigates perioperative neurocognitive disorders by regulating the function of the glymphatic system. Cell Commun Signal. (2026) 24:164. doi: 10.1186/s12964-026-02751-0. PMID: 41803833 PMC12973863

[138] Chen K Du X Chao MA Xie Z Yang G . Surgery impairs glymphatic activity and cognitive function in aged mice. Mol Brain. (2025) 18:7. doi: 10.1186/s13041-025-01177-y. PMID: 39856767 PMC11763125

[139] Yu Y Liu X Zang Z Zhao X Zhao B Zhang Y . Enhanced meningeal lymphatic drainage alleviates cognitive dysfunction induced by anesthesia and surgery in aged mice. Neuropharmacology. (2025) 280:110674. doi: 10.1016/j.neuropharm.2025.110674. PMID: 40902879

[140] Zhu X Lin J Yang P Wu S Lin H He W . Surgery induces neurocognitive disorder via neuroinflammation and glymphatic dysfunction in middle-aged mice with brain lymphatic drainage impairment. Front Neurosci. (2024) 18:1426718. doi: 10.3389/fnins.2024.1426718. PMID: 38975244 PMC11225229

[141] VuEL BrownCH BradyKM HogueCW . Monitoring of cerebral blood flow autoregulation: physiologic basis, measurement, and clinical implications. Br J Anaesth. (2024) 132:1260–73. doi: 10.1016/j.bja.2024.01.043. PMID: 38471987

[142] Claesson-Lingehall H Olofsson B Gustafson Y Wahba A Appelblad M Svenmarker S . Hemodynamic control during cardiopulmonary bypass and the incidence of postoperative delirium- a post hoc analysis. BMC Anesthesiol. (2025) 25:267. doi: 10.1186/s12871-025-03141-8. PMID: 40419968 PMC12105260

[143] Han G Jiao B Zhang Y Wang Z Liang C Li Y . Arterial pulsation dependence of perivascular cerebrospinal fluid flow measured by dynamic diffusion tensor imaging in the human brain. Neuroimage. (2024) 297:120653. doi: 10.1016/j.neuroimage.2024.120653. PMID: 38795798

[144] Zimmermann J Sorg C Müller L Zistler F Neumaier V Bonhoeffer M . Impaired macroscopic cerebrospinal fluid flow by Sevoflurane in humans during and after anesthesia. Anesthesiology. (2025) 142:692–703. doi: 10.1097/ALN.0000000000005360. PMID: 39786916 PMC11893005

[145] Abdelraouf MR Mahmoud A Amin AM Salamah HM Alshaker H Rezq H . The impact of pulsatile vs. non-pulsatile perfusion in patients undergoing cardiopulmonary bypass: a comprehensive systematic review and meta-analysis of 33 randomized controlled trials. PloS One. (2025) 20:e0333495. doi: 10.1371/journal.pone.0333495. PMID: 41086150 PMC12520390

[146] Scott DHT . Non-invasive blood pressure measurement displays. Anaesthesia. (2018) 73:1299. doi: 10.1111/anae.14433. PMID: 30216427

[147] Stanton EH Persson NDÅ Gomolka RS Lilius T Sigurðsson B Lee H . Mapping of CSF transport using high spatiotemporal resolution dynamic contrast-enhanced MRI in mice: effect of anesthesia. Magn Reson Med. (2021) 85:3326–42. doi: 10.1002/mrm.28645. PMID: 33426699

[148] Kılıç K Devor A . The stop and go of glymphatic flow. Nat Neurosci. (2023) 26:924–5. doi: 10.1038/s41593-023-01344-1. PMID: 37264157

[149] Liu G Mestre H Sweeney AM Sun Q Weikop P Du T . Direct measurement of cerebrospinal fluid production in mice. Cell Rep. (2020) 33:108524. doi: 10.1016/j.celrep.2020.108524. PMID: 33357428 PMC8186543

[150] Maneshi MM Maki B Gnanasambandam R Belin S Popescu GK Sachs F . Mechanical stress activates NMDA receptors in the absence of agonists. Sci Rep. (2017) 7:39610. doi: 10.1038/srep39610. PMID: 28045032 PMC5206744

[151] Duman RS Shinohara R Fogaça MV Hare B . Neurobiology of rapid-acting antidepressants: convergent effects on GluA1-synaptic function. Mol Psychiatry. (2019) 24:1816–32. doi: 10.1038/s41380-019-0400-x. PMID: 30894661 PMC6754322

[152] Zhou B Chen L Liao P Huang L Chen Z Liao D . Astroglial dysfunctions drive aberrant synaptogenesis and social behavioral deficits in mice with neonatal exposure to lengthy general anesthesia. PloS Biol. (2019) 17:e3000086. doi: 10.1371/journal.pbio.3000086. PMID: 31433818 PMC6719896

[153] Hattori JI Yamakage M Seki S Okazaki K Namiki A . Inhibitory effects of the anesthetics propofol and sevoflurane on spontaneous lymphatic vessel activity in rats. Anesthesiology. (2004) 101:687–94. doi: 10.1097/00000542-200409000-00017. PMID: 15329593

[154] Jo AO Ryskamp DA Phuong TTT Verkman AS Yarishkin O MacAulay N . TRPV4 and AQP4 channels synergistically regulate cell volume and calcium homeostasis in retinal müller glia. J Neurosci. (2015) 35:13525–37. doi: 10.1523/JNEUROSCI.1987-15.2015. PMID: 26424896 PMC4588615

[155] Ou M Kuo FS Chen X Kahanovitch U Olsen ML Du G . Isoflurane inhibits a Kir4.1/5.1-like conductance in neonatal rat brainstem astrocytes and recombinant kir4.1/5.1 channels in a heterologous expression system. J Neurophysiol. (2020) 124:740–9. doi: 10.1152/jn.00358.2020. PMID: 32727273 PMC7509298

[156] Wang Y Huang C Guo Q Chu H . Aquaporin-4 and cognitive disorders. Aging Dis. (2022) 13:61–72. doi: 10.14336/AD.2021.0731. PMID: 35111362 PMC8782559

[157] Guo Y Wu L Liu J Liu J Sun Z . Correlation between glymphatic dysfunction and cranial defect in severe traumatic brain injury: a retrospective case-control study based on a diffusion tensor image analysis along the perivascular space (DTI-ALPS) investigation. Quant Imaging Med Surg. (2024) 14:6756–66. doi: 10.21037/qims-24-348. PMID: 39281142 PMC11400707

[158] Schartz D Finkelstein A Bender M Kessler A Zhong J . Association of extent of transverse sinus stenosis with cerebral glymphatic clearance in patients with idiopathic intracranial hypertension. Neurology. (2024) 103:e209529. doi: 10.1212/WNL.0000000000209529. PMID: 38833652

[159] Hauglund NL Kusk P Kornum BR Nedergaard M . Meningeal lymphangiogenesis and enhanced glymphatic activity in mice with chronically implanted EEG electrodes. J Neurosci. (2020) 40:2371–80. doi: 10.1523/JNEUROSCI.2223-19.2020. PMID: 32047056 PMC7083292

[160] Zou K Deng Q Zhang H Huang C . Glymphatic system: a gateway for neuroinflammation. Neural Regener Res. (2023) 19:2661–72. doi: 10.4103/1673-5374.391312. PMID: 38595285 PMC11168510

[161] Da Mesquita S Papadopoulos Z Dykstra T Brase L Farias FG Wall M . Meningeal lymphatics affect microglia responses and anti-Aβ immunotherapy. Nature. (2021) 593:255–60. doi: 10.1038/s41586-021-03489-0. PMID: 33911285 PMC8817786

[162] Drieu A Du S Kipnis M Bosch ME Herz J Lee C . Parenchymal border macrophages regulate tau pathology and tau-mediated neurodegeneration. Life Sci All. (2023) 6:e202302087. doi: 10.26508/lsa.202302087. PMID: 37562846 PMC10415611

[163] Zhou Y Xue R Li Y Ran W Chen Y Luo Z . Impaired meningeal lymphatics and glymphatic pathway in patients with white matter hyperintensity. Adv Sci. (2024) 11:e2402059. doi: 10.1002/advs.202402059. PMID: 38704728 PMC11234435

[164] Liu J Gao D Hu D Lan S Liu Y Zheng H . Delivery of biomimetic liposomes via meningeal lymphatic vessels route for targeted therapy of Parkinson’s disease. Research. (2023) 6:30. doi: 10.34133/research.0030. PMID: 37040500 PMC10076012

[165] Shang P Zheng R Wu K Yuan C Pan S . New insights on mechanisms and therapeutic targets of cerebral edema. Curr Neuropharmacol. (2024) 22:2330–52. doi: 10.2174/1570159X22666240528160237. PMID: 38808718 PMC11451312

[166] Perla M Caretti V Moro MA McCullough LD . Role of the meningeal lymphatics in stroke. Stroke. (2023) 54:1670–3. doi: 10.1161/STROKEAHA.123.043424. PMID: 37216448 PMC10204316

[167] Wu W Zhao Y Cheng X Xie X Zeng Y Tao Q . Modulation of glymphatic system by visual circuit activation alleviates memory impairment and apathy in a mouse model of Alzheimer’s disease. Nat Commun. (2025) 16:63. doi: 10.1038/s41467-024-55678-w. PMID: 39747869 PMC11696061

[168] Bolte AC Dutta AB Hurt ME Smirnov I Kovacs MA McKee CA . Meningeal lymphatic dysfunction exacerbates traumatic brain injury pathogenesis. Nat Commun. (2020) 11:4524. doi: 10.1038/s41467-020-18113-4. PMID: 32913280 PMC7483525

[169] Liao J Zhang M Shi Z Lu H Wang L Fan W . Improving the function of meningeal lymphatic vessels to promote brain edema absorption after traumatic brain injury. J Neurotrauma. (2023) 40:383–94. doi: 10.1089/neu.2022.0150. PMID: 36106596

[170] Holstein-Rønsbo S Gan Y Giannetto MJ Rasmussen MK Sigurdsson B Beinlich FRM . Glymphatic influx and clearance are accelerated by neurovascular coupling. Nat Neurosci. (2023) 26:1042–53. doi: 10.1038/s41593-023-01327-2. PMID: 37264158 PMC10500159

[171] Evered L Silbert B Scott DA Ames D Maruff P Blennow K . Cerebrospinal fluid biomarker for Alzheimer disease predicts postoperative cognitive dysfunction. Anesthesiology. (2016) 124:353–61. doi: 10.1097/ALN.0000000000000953. PMID: 26580833

[172] Gerlach RM Chaney MA . Postoperative cognitive dysfunction related to Alzheimer disease? J Thorac Cardiovasc Surg. (2018) 155:968–9. doi: 10.1016/j.jtcvs.2017.10.113. PMID: 29336803

[173] Evered L Silbert B Scott DA Zetterberg H Blennow K . Association of changes in plasma neurofilament light and tau levels with anesthesia and surgery: results from the CAPACITY and ARCADIAN studies. JAMA Neurol. (2018) 75:542. doi: 10.1001/jamaneurol.2017.4913. PMID: 29459944 PMC5885271

[174] Zhou Y Wang J Li X Li K Chen L Zhang Z . Neuroprotectin d1 protects against postoperative delirium-like behavior in aged mice. Front Aging Neurosci. (2020) 12:582674. doi: 10.3389/fnagi.2020.582674. PMID: 33250764 PMC7674198

[175] Liddelow SA Guttenplan KA Clarke LE Bennett FC Bohlen CJ Schirmer L . Neurotoxic reactive astrocytes are induced by activated microglia. Nature. (2017) 541:481–7. doi: 10.1038/nature21029. PMID: 28099414 PMC5404890

[176] Da Mesquita S Herz J Wall M Dykstra T de Lima KA Norris GT . Aging-associated deficit in CCR7 is linked to worsened glymphatic function, cognition, neuroinflammation, and β-amyloid pathology. Sci Adv. (2021) 7:eabe4601. doi: 10.1126/sciadv.abe4601. PMID: 34020948 PMC8139596

[177] Wang LY Wang XP Lv JM Shan YD Jia SY Yu ZF . NLRP3-GABA signaling pathway contributes to the pathogenesis of impulsive-like behaviors and cognitive deficits in aged mice. J Neuroinflamm. (2023) 20:162. doi: 10.1186/s12974-023-02845-3. PMID: 37434240 PMC10337164

[178] Zang X Chen S Zhu J Ma J Zhai Y . The emerging role of central and peripheral immune systems in neurodegenerative diseases. Front Aging Neurosci. (2022) 14:872134. doi: 10.3389/fnagi.2022.872134. PMID: 35547626 PMC9082639

[179] Thompson D Brissette CA Watt JA . The choroid plexus and its role in the pathogenesis of neurological infections. Fluid Bar CNS. (2022) 19:75. doi: 10.1186/s12987-022-00372-6. PMID: 36088417 PMC9463972

[180] Jin H Li M Jeong E Castro-Martinez F Zuker CS . A body–brain circuit that regulates body inflammatory responses. Nature. (2024) 630:695–703. doi: 10.1038/s41586-024-07469-y. PMID: 38692285 PMC11186780

[181] Miyata S . Glial functions in the blood-brain communication at the circumventricular organs. Front Neurosci. (2022) 16:991779. doi: 10.3389/fnins.2022.991779. PMID: 36278020 PMC9583022

[182] Saunders NR Dziegielewska KM Fame RM Lehtinen MK Liddelow SA . The choroid plexus: a missing link in our understanding of brain development and function. Physiol Rev. (2023) 103:919–56. doi: 10.1152/physrev.00060.2021. PMID: 36173801 PMC9678431

[183] Johanson C Stopa E McMillan P Roth D Funk J Krinke G . The distributional nexus of choroid plexus to cerebrospinal fluid, ependyma and brain: toxicologic/pathologic phenomena, periventricular destabilization, and lesion spread. Toxicol Pathol. (2011) 39:186–212. doi: 10.1177/0192623310394214. PMID: 21189316

[184] Denver P Tortorelli L Hov K Berg JP Giil LM Nazmi A . Chemokine associations with blood cerebrospinal fluid (CSF) barrier permeability and delirium. Brain Behav Immun Health. (2025) 43:100920. doi: 10.1016/j.bbih.2024.100920. PMID: 39839987 PMC11750293

[185] Xu H Lotfy P Gelb S Pragana A Hehnly C Byer LIJ . The choroid plexus synergizes with immune cells during neuroinflammation. Cell. (2024) 187:4946–4963.e17. doi: 10.1016/j.cell.2024.07.002. PMID: 39089253 PMC11458255

[186] Hochstetler A Lehtinen MK . Choroid plexus as a mediator of CNS inflammation in multiple sclerosis. Mult Scler. (2024) 30:19–23. doi: 10.1177/13524585241292974. PMID: 39503321 PMC11634642

[187] Xiong C Liu J Lin D Zhang J Terrando N Wu A . Complement activation contributes to perioperative neurocognitive disorders in mice. J Neuroinflamm. (2018) 15:254. doi: 10.1186/s12974-018-1292-4. PMID: 30180861 PMC6123969

[188] Madadi AK Sohn MJ . Advances in intrathecal nanoparticle delivery: targeting the blood-cerebrospinal fluid barrier for enhanced CNS drug delivery. Pharmaceuticals. (2024) 17:1070. doi: 10.3390/ph17081070. PMID: 39204177 PMC11357388

[189] Loeffler DA . Enhancing of cerebral Abeta clearance by modulation of ABC transporter expression: a review of experimental approaches. Front Aging Neurosci. (2024) 16:1368200. doi: 10.3389/fnagi.2024.1368200. PMID: 38872626 PMC11170721

[190] Suzuki Y Nakamura Y Igarashi H . Blood cerebrospinal fluid barrier function disturbance can be followed by amyloid-β accumulation. J Clin Med. (2022) 11:6118. doi: 10.3390/jcm11206118. PMID: 36294439 PMC9605218

[191] Zhu HH Li SS Wang YC Song B Gao Y Xu YM . Clearance dysfunction of trans-barrier transport and lymphatic drainage in cerebral small vessel disease: review and prospect. Neurobiol Dis. (2023) 189:106347. doi: 10.1016/j.nbd.2023.106347. PMID: 37951367

[192] Louveau A Plog BA Antila S Alitalo K Nedergaard M Kipnis J . Understanding the functions and relationships of the glymphatic system and meningeal lymphatics. J Clin Invest. (2017) 127:3210–9. doi: 10.1172/JCI90603. PMID: 28862640 PMC5669566

[193] Mogensen FLH Delle C Nedergaard M . The glymphatic system (en)during inflammation. Int J Mol Sci. (2021) 22:7491. doi: 10.3390/ijms22147491. PMID: 34299111 PMC8305763

[194] Benarroch EE . Circumventricular organs: receptive and homeostatic functions and clinical implications. Neurology. (2011) 77:1198–204. doi: 10.1212/WNL.0b013e31822f04a0. PMID: 21931109

[195] Wei SG Zhang ZH Beltz TG Yu Y Johnson AK Felder RB . Subfornical organ mediates sympathetic and hemodynamic responses to blood-borne proinflammatory cytokines. Hypertension. (2013) 62:118–25. doi: 10.1161/HYPERTENSIONAHA.113.01404. PMID: 23670302 PMC3769944

[196] Korim WS Elsaafien K Basser JR Setiadi A May CN Yao ST . In renovascular hypertension, TNF-α type-1 receptors in the area postrema mediate increases in cardiac and renal sympathetic nerve activity and blood pressure. Cardiovasc Res. (2019) 115:1092–101. doi: 10.1093/cvr/cvy268. PMID: 30358805

[197] Okamoto A Fujii R Yoshimura R Miyata S . Transcytosis of tanycytes in the circumventricular organs of adult mouse brain. Neurosci Lett. (2022) 779:136633. doi: 10.1016/j.neulet.2022.136633. PMID: 35429588

[198] Jhang SY Lee SH Lee EB Choi JH Bang S Jeong M . Effects of platycodon grandiflorum on gut microbiome and immune system of immunosuppressed mouse. Metabolites. (2021) 11:817. doi: 10.3390/metabo11120817. PMID: 34940575 PMC8707369

[199] Schulz M Engelhardt B . The circumventricular organs participate in the immunopathogenesis of experimental autoimmune encephalomyelitis. Cerebrospinal Fluid Res. (2005) 2:8. doi: 10.1186/1743-8454-2-8. PMID: 16197544 PMC1262737

[200] Manouchehrian O Ramos M Bachiller S Lundgaard I Deierborg T . Acute systemic LPS-exposure impairs perivascular CSF distribution in mice. J Neuroinflamm. (2021) 18:34. doi: 10.1186/s12974-021-02082-6. PMID: 33514389 PMC7844902

[201] Hillebrand S Schanda K Nigritinou M Tsymala I Böhm D Peschl P . Circulating AQP4-specific auto-antibodies alone can induce neuromyelitis optica spectrum disorder in the rat. Acta Neuropathol. (2019) 137:467–85. doi: 10.1007/s00401-018-1950-8. PMID: 30564980 PMC6514074

[202] Berthoud HR Neuhuber WL . Functional and chemical anatomy of the afferent vagal system. Auton Neurosci. (2000) 85:1–17. doi: 10.1016/S1566-0702(00)00215-0. PMID: 11189015

[203] Hwang YK Oh JS . Interaction of the vagus nerve and serotonin in the gut-brain axis. Int J Mol Sci. (2025) 26:1160. doi: 10.3390/ijms26031160. PMID: 39940928 PMC11818468

[204] Trevizan-Baú P McAllen RM . What is the vagal-adrenal axis? J Comp Neurol. (2024) 532:e25656. doi: 10.1002/cne.25656. PMID: 38980012

[205] Davis EA Wald HS Suarez AN Zubcevic J Liu CM Cortella AM . Ghrelin signaling affects feeding behavior, metabolism, and memory through the vagus nerve. Curr Biol. (2020) 30:4510–4518.e6. doi: 10.1016/j.cub.2020.08.069. PMID: 32946754 PMC7674191

[206] Sun L Peng XL Zi HX Zhang ZK Gong YC Li J . Globally patterned locus coeruleus-norepinephrine neuron-pericyte coupling orchestrates brain-wide vascular dynamics. Neuron. (2026) 114:287–306.e9. doi: 10.1016/j.neuron.2025.10.010. PMID: 41232533

[207] Cox TO Devason AS de Araujo A Mason S Subramanian M Salvador AFM . Intestinal interoceptive dysfunction drives age-associated cognitive decline. Nature. (2026) 652:442–450. doi: 10.1038/s41586-026-10191-6. PMID: 41813891 PMC13061634

[208] Pan Z Jia Z Jiang T Cai Q Di Z Gan L . Modulation of the neurovascular unit by the locus coeruleus-norepinephrine system: from physiological mechanisms to therapeutic applications. FASEB J. (2025) 39:e71127. doi: 10.1096/fj.202502069R. PMID: 41078309 PMC12516802

[209] Choi S Baek IS Lee K Kim SK . Low-frequency auricular vagus nerve stimulation facilitates cerebrospinal fluid influx by promoting vasomotion. Kor J Physiol Pharmacol. (2025) 29:109–16. doi: 10.4196/kjpp.24.266. PMID: 39482237 PMC11694001

[210] Cheng KP Brodnick SK Blanz SL Zeng W Kegel J Pisaniello JA . Clinically-derived vagus nerve stimulation enhances cerebrospinal fluid penetrance. Brain Stimulat. (2020) 13:1024–30. doi: 10.1016/j.brs.2020.03.012. PMID: 32388045

[211] Ghersi-Egea JF Strazielle N Catala M Silva-Vargas V Doetsch F Engelhardt B . Molecular anatomy and functions of the choroidal blood-cerebrospinal fluid barrier in health and disease. Acta Neuropathol. (2018) 135:337–61. doi: 10.1007/s00401-018-1807-1. PMID: 29368213

[212] Braun M Iliff JJ . The impact of neurovascular, blood-brain barrier, and glymphatic dysfunction in neurodegenerative and metabolic diseases. Int Rev Neurobiol. (2020) 154:413–36. doi: 10.1016/bs.irn.2020.02.006. PMID: 32739013

[213] Municio C Carrero L Antequera D Carro E . Choroid plexus aquaporins in CSF homeostasis and the glymphatic system: their relevance for Alzheimer’s disease. Int J Mol Sci. (2023) 24:878. doi: 10.3390/ijms24010878. PMID: 36614315 PMC9821203

[214] Olsen LK Solis E McIntire LK Hatcher-Solis CN . Vagus nerve stimulation: mechanisms and factors involved in memory enhancement. Front Hum Neurosci. (2023) 17:1152064. doi: 10.3389/fnhum.2023.1152064. PMID: 37457500 PMC10342206

[215] Yamada S Wang Y Monai H . Transcranial cortex-wide Ca^2+^ imaging for the functional mapping of cortical dynamics. Front Neurosci. (2023) 17:1119793. doi: 10.3389/fnins.2023.1119793. PMID: 36875638 PMC9975744

[216] Bèchet NB Shanbhag NC Lundgaard I . Glymphatic pathways in the gyrencephalic brain. J Cereb Blood Flow Metab. (2021) 41:2264–79. doi: 10.1177/0271678X21996175. PMID: 33641515 PMC8393296

[217] Bèchet NB Kylkilahti TM Mattsson B Petrasova M Shanbhag NC Lundgaard I . Light sheet fluorescence microscopy of optically cleared brains for studying the glymphatic system. J Cereb Blood Flow Metab. (2020) 40:1975–86. doi: 10.1177/0271678X20924954. PMID: 32525440 PMC7786847

[218] Wang Z Yang F Shi W Xie W Zhang Z Yang S . Monitoring the perivascular cerebrospinal fluid dynamics of the glymphatic pathway using co-localized photoacoustic microscopy. Opt Lett. (2023) 48:2265–8. doi: 10.1364/OL.486129. PMID: 37126250

[219] Jacob L Brito J Thomas JL . Three-dimensional imaging of the vertebral lymphatic vasculature and drainage using iDISCO+ and light sheet fluorescence microscopy. J Vis Exp. (2020), (159). doi: 10.3791/61099. PMID: 32510513

[220] Wang C Su D Zhang Z Chen J Liu Y Peng C . Zebrafish fluorescence imaging platform based on bessel light sheet illumination. BioMed Opt Express. (2025) 16:1678–91. doi: 10.1364/BOE.542599. PMID: 40321999 PMC12047710

[221] Xue Y Liu X Koundal S Constantinou S Dai F Santambrogio L . *In vivo* T1 mapping for quantifying glymphatic system transport and cervical lymph node drainage. Sci Rep. (2020) 10:14592. doi: 10.1038/s41598-020-71582-x. PMID: 32884041 PMC7471332

[222] Lyu C Xia Y Li Y Que J Han F Guan Q . Dynamic contrast-enhanced MRI reveals glymphatic dysfunction in mice with depressive-like behavior. Neurobiol Dis. (2025) 217:107169. doi: 10.1016/j.nbd.2025.107169. PMID: 41205656

[223] Bai Y Yuan M Mi H Zhang F Liu X Lu C . Hypothermia reduces glymphatic transportation in traumatic edematous brain assessed by intrathecal dynamic contrast-enhanced MRI. Front Neurol. (2022) 13:957055. doi: 10.3389/fneur.2022.957055. PMID: 36341130 PMC9632734

[224] Zhu Y Wang G Kolluru C Gu Y Gao H Zhang J . Transport pathways and kinetics of cerebrospinal fluid tracers in mouse brain observed by dynamic contrast-enhanced MRI. Sci Rep. (2023) 13:13882. doi: 10.1038/s41598-023-40896-x. PMID: 37620371 PMC10449788

[225] Ringstad G Valnes LM Dale AM Pripp AH Vatnehol SAS Emblem KE . Brain-wide glymphatic enhancement and clearance in humans assessed with MRI. JCI Insight. (2018) 3:e121537. doi: 10.1172/jci.insight.121537. PMID: 29997300 PMC6124518

[226] Deike-Hofmann K Reuter J Haase R Paech D Gnirs R Bickelhaupt S . Glymphatic pathway of gadolinium-based contrast agents through the brain: overlooked and misinterpreted. Invest Radiol. (2019) 54:229–37. doi: 10.1097/RLI.0000000000000533. PMID: 30480554

[227] Ding G Chopp M Li L Zhang L Davoodi-Bojd E Li Q . MRI investigation of glymphatic responses to Gd-DTPA infusion rates. J Neurosci Res. (2018) 96:1876–86. doi: 10.1002/jnr.24325. PMID: 30272825 PMC6186187

[228] Kiviniemi V Wang X Korhonen V Keinänen T Tuovinen T Autio J . Ultra-fast magnetic resonance encephalography of physiological brain activity - glymphatic pulsation mechanisms? J Cereb Blood Flow Metab. (2016) 36:1033–45. doi: 10.1177/0271678X15622047. PMID: 26690495 PMC4908626

[229] Naganawa S Taoka T . The glymphatic system: a review of the challenges in visualizing its structure and function with MR imaging. Magn Reson Med Sci. (2022) 21:182–94. doi: 10.2463/mrms.rev.2020-0122. PMID: 33250472 PMC9199971

[230] Kim JH Im JG Park SH . Measurement of CSF pulsation from EPI-based human fMRI. Neuroimage. (2022) 257:119293. doi: 10.1016/j.neuroimage.2022.119293. PMID: 35551990

[231] Kim J Lipford ME Barcus RA Frye BM Yuan H Lyu Q . Effects of diminished cerebrospinal fluid flow in the spinal canal on amyloid pathophysiology in vervet monkeys. In: Research square (Durham, NC: Research Square) (2025). doi: 10.21203/rs.3.rs-6551242/v1, PMID:

[232] Park M Kim JW Ahn SJ Cha YJ Suh SH . Aging is positively associated with peri-sinus lymphatic space volume: assessment using 3T black-blood MRI. J Clin Med. (2020) 9:3353. doi: 10.3390/jcm9103353. PMID: 33086702 PMC7590154

[233] Calabro FJ Parr AC Sydnor VJ Hetherington H Prasad KM Ibrahim TS . Leveraging ultra-high field (7T) MRI in psychiatric research. Neuropsychopharmacology. (2024) 50:85–102. doi: 10.1038/s41386-024-01980-6. PMID: 39251774 PMC11525672

[234] Neuner I Veselinović T Ramkiran S Rajkumar R Schnellbaecher GJ Shah NJ . 7T ultra-high-field neuroimaging for mental health: an emerging tool for precision psychiatry? Transl Psychiatry. (2022) 12:36. doi: 10.1038/s41398-022-01787-3. PMID: 35082273 PMC8791951

[235] Patel LD Raghavan P Tang S Choi S Harrison DM . Imaging of the meningeal lymphatic network in healthy adults: a 7T MRI study. J Neuroradiol. (2023) 50:369–76. doi: 10.1016/j.neurad.2023.03.002. PMID: 36918053 PMC10981496

[236] Hildesheim FE Ramasamy DP Bergsland N Jakimovski D Dwyer MG Hojnacki D . Leptomeningeal, dura mater and meningeal vessel wall enhancements in multiple sclerosis. Mult Scler Relat Disord. (2021) 47:102653. doi: 10.1016/j.msard.2020.102653. PMID: 33333417

[237] Filippopulos FM Fischer TD Seelos K Dunker K Belanovic B Crispin A . Semiquantitative 3T brain magnetic resonance imaging for dynamic visualization of the glymphatic-lymphatic fluid transport system in humans: a pilot study. Invest Radiol. (2022) 57:544–51. doi: 10.1097/RLI.0000000000000870. PMID: 35763443

[238] Myllylä T Harju M Korhonen V Bykov A Kiviniemi V Meglinski I . Assessment of the dynamics of human glymphatic system by near-infrared spectroscopy. J Biophoton. (2018) 11:e201700123. doi: 10.1002/jbio.201700123. PMID: 28802090

[239] Yoon JE Ji M Hwang I Lee WJ Yu S Kim J . Brain water dynamics across sleep stages measured by near-infrared spectroscopy: implications for glymphatic function. J Cereb Blood Flow Metab. (2025) 45:2203–16. doi: 10.1177/0271678X251353142. PMID: 40562709 PMC12202386

[240] Lilius TO Rosenholm M Klinger L Mortensen KN Sigurdsson B Mogensen FLH . SPECT/CT imaging reveals CNS-wide modulation of glymphatic cerebrospinal fluid flow by systemic hypertonic saline. iScience. (2022) 25:105250. doi: 10.1016/j.isci.2022.105250. PMID: 36274948 PMC9579504

[241] Qin Y He R Chen J Zhou X Zhou X Liu Z . Neuroimaging uncovers distinct relationships of glymphatic dysfunction and motor symptoms in Parkinson’s disease. J Neurol. (2023) 270:2649–58. doi: 10.1007/s00415-023-11594-5. PMID: 36856846

[242] Kroesbergen E Riesselmann LV Gomolka RS Plá V Esmail T Stenmo VH . Glymphatic clearance is enhanced during sleep. In: BioRxiv prepr serv biol (Cold Spring Harbor, NY: Cold Spring Harbor Laboratory). (2024). p. 2024.08.24.609514. doi: 10.1101/2024.08.24.609514, PMID:

[243] Benveniste H Lee H Ozturk B Chen X Koundal S Vaska P . Glymphatic cerebrospinal fluid and solute transport quantified by MRI and PET imaging. Neuroscience. (2021) 474:63–79. doi: 10.1016/j.neuroscience.2020.11.014. PMID: 33248153 PMC8149482

[244] Van Hove H Martens L Scheyltjens I De Vlaminck K Pombo Antunes AR De Prijck S . A single-cell atlas of mouse brain macrophages reveals unique transcriptional identities shaped by ontogeny and tissue environment. Nat Neurosci. (2019) 22:1021–35. doi: 10.1038/s41593-019-0393-4. PMID: 31061494

[245] Quintana JF Sinton MC Chandrasegaran P Kumar Dubey L Ogunsola J Al Samman M . The murine meninges acquire lymphoid tissue properties and harbour autoreactive B cells during chronic Trypanosoma brucei infection. PloS Biol. (2023) 21:e3002389. doi: 10.1371/journal.pbio.3002389. PMID: 37983289 PMC10723712

[246] Rappold T Laflam A Hori D Brown C Brandt J Mintz CD . Evidence of an association between brain cellular injury and cognitive decline after non-cardiac surgery. Br J Anaesth. (2016) 116:83–9. doi: 10.1093/bja/aev415. PMID: 26675953 PMC4681618

[247] Hall RJ Ferguson KJ Andrews M Green AJE White TO Armstrong IR . Delirium and cerebrospinal fluid S100B in hip fracture patients: a preliminary study. Am J Geriatr Psychiatry. (2013) 21:1239–43. doi: 10.1016/j.jagp.2012.12.024. PMID: 23602305

[248] Benedet AL Milà-Alomà M Vrillon A Ashton NJ Pascoal TA Lussier F . Differences between plasma and cerebrospinal fluid glial fibrillary acidic protein levels across the Alzheimer disease continuum. JAMA Neurol. (2021) 78:1471–83. doi: 10.1001/jamaneurol.2021.3671. PMID: 34661615 PMC8524356

[249] Henjum K Quist-Paulsen E Zetterberg H Blennow K Nilsson LNG Watne LO . CSF sTREM2 in delirium-relation to Alzheimer’s disease CSF biomarkers Aβ42, t-tau and p-tau. J Neuroinflamm. (2018) 15:304. doi: 10.1186/s12974-018-1331-1. PMID: 30390679 PMC6215363

[250] Hablitz LM Vinitsky HS Sun Q Stæger FF Sigurdsson B Mortensen KN . Increased glymphatic influx is correlated with high EEG delta power and low heart rate in mice under anesthesia. Sci Adv. (2019) 5:eaav5447. doi: 10.1126/sciadv.aav5447. PMID: 30820460 PMC6392807

[251] Evered LA Goldstein PA . Reducing perioperative neurocognitive disorders (PND) through depth of anesthesia monitoring: a critical review. Int J Gen Med. (2021) 14:153–62. doi: 10.2147/IJGM.S242230. PMID: 33469352 PMC7813450

[252] Groothuis DR Vavra MW Schlageter KE Kang EWY Itskovich AC Hertzler S . Efflux of drugs and solutes from brain: the interactive roles of diffusional transcapillary transport, bulk flow and capillary transporters. J Cereb Blood Flow Metab. (2007) 27:43–56. doi: 10.1038/sj.jcbfm.9600315. PMID: 16639426

[253] von Holstein-Rathlou S Petersen NC Nedergaard M . Voluntary running enhances glymphatic influx in awake behaving, young mice. Neurosci Lett. (2018) 662:253–8. doi: 10.1016/j.neulet.2017.10.035. PMID: 29079431 PMC5696653

[254] Dong R Liu W Han Y Wang Z Jiang L Wang L . Influencing factors of glymphatic system during perioperative period. Front Neurosci. (2024) 18:1428085. doi: 10.3389/fnins.2024.1428085. PMID: 39328423 PMC11424614

[255] DiNuzzo M Nedergaard M . Brain energetics during the sleep-wake cycle. Curr Opin Neurobiol. (2017) 47:65–72. doi: 10.1016/j.conb.2017.09.010. PMID: 29024871 PMC5732842

[256] Lilius TO Blomqvist K Hauglund NL Liu G Stæger FF Bærentzen S . Dexmedetomidine enhances glymphatic brain delivery of intrathecally administered drugs. J Ctrl Release. (2019) 304:29–38. doi: 10.1016/j.jconrel.2019.05.005. PMID: 31067483

[257] Benveniste H Lee H Ding F Sun Q Al-Bizri E Makaryus R . Anesthesia with Dexmedetomidine and low-dose Isoflurane increases solute transport via the glymphatic pathway in rat brain when compared with high-dose Isoflurane. Anesthesiology. (2017) 127:976–88. doi: 10.1097/ALN.0000000000001888. PMID: 28938276 PMC5685871

[258] Ozturk BO Monte B Koundal S Dai F Benveniste H Lee H . Disparate volumetric fluid shifts across cerebral tissue compartments with two different anesthetics. Fluid Bar CNS. (2021) 18:1. doi: 10.1186/s12987-020-00236-x. PMID: 33407650 PMC7788828

[259] León-Salas B Trujillo-Martín MM Del Castillo LPM García JG Pérez-Ros P Ruiz FR . Pharmacologic interventions for prevention of delirium in hospitalized older people: a meta-analysis. Arch Gerontol Geriatr. (2020) 90:104171. doi: 10.1016/j.archger.2020.104171. PMID: 32682169

[260] Pieri M De Simone A Rose S De Domenico P Lembo R Denaro G . Trials focusing on prevention and treatment of delirium after cardiac surgery: a systematic review of randomized evidence. J Cardiothorac Vasc Anesth. (2020) 34:1641–54. doi: 10.1053/j.jvca.2019.09.028. PMID: 31668634

[261] O’Kane A Quinney SK Kinney E Bergstrom RF Tillman EM . A systematic review of dexmedetomidine pharmacology in pediatric patients. Clin Transl Sci. (2024) 17:e70020. doi: 10.1111/cts.70020. PMID: 39644147 PMC11624482

[262] Gakuba C Gaberel T Goursaud S Bourges J Di Palma C Quenault A . General anesthesia inhibits the activity of the "glymphatic system. Theranostics. (2018) 8:710–22. doi: 10.7150/thno.19154. PMID: 29344300 PMC5771087

[263] Taoka T Jost G Frenzel T Naganawa S Pietsch H . Impact of the glymphatic system on the kinetic and distribution of gadodiamide in the rat brain: observations by dynamic MRI and effect of circadian rhythm on tissue gadolinium concentrations. Invest Radiol. (2018) 53:529–34. doi: 10.1097/RLI.0000000000000473. PMID: 29652699

[264] Liu H Yang C Wang X Yu B Han Y Wang X . Propofol improves sleep deprivation-induced sleep structural and cognitive deficits via upregulating the BMAL1 expression and suppressing microglial M1 polarization. CNS Neurosci Ther. (2024) 30:e14798. doi: 10.1111/cns.14798. PMID: 39015099 PMC11252557

[265] Ding X Lu Y Chen J Chen X . Propofol attenuates LPS-induced inflammation by suppressing the activation of histone lactylation in hCMEC/D3 cells. Curr Neurovasc Res. (2025) 22:333–45. doi: 10.2174/0115672026423091251211090557. PMID: 41479382

[266] Ma Q Ries M Decker Y Müller A Riner C Bücker A . Rapid lymphatic efflux limits cerebrospinal fluid flow to the brain. Acta Neuropathol. (2019) 137:151–65. doi: 10.1007/s00401-018-1916-x. PMID: 30306266 PMC6338719

[267] Kim HY Dhong HJ Lee JK Chung SK Jung SC . Sleep quality and effects of position on sleep apnea in East Asian children. Auris Nasus Larynx. (2011) 38:228–32. doi: 10.1016/j.anl.2010.07.005. PMID: 20800981

[268] Eide PK Hovd M Pripp A Gjertsen Ø Løvland G Lashkarivand A . Altered brain fluid dynamics in spontaneous intracranial hypotension. Fluid Bar CNS. (2025) 22:65. doi: 10.1186/s12987-025-00679-0. PMID: 40597337 PMC12210607

[269] van Hattem T Verkaar L Krugliakova E Adelhöfer N Zeising M Drinkenburg WHIM . Targeting sleep physiology to modulate glymphatic brain clearance. Physiology. (2025) 40:271–90. doi: 10.1152/physiol.00019.2024. PMID: 39601891

[270] Cai X Qiao J Kulkarni P Harding IC Ebong E Ferris CF . Imaging the effect of the circadian light-dark cycle on the glymphatic system in awake rats. Proc Natl Acad Sci USA. (2020) 117:668–76. doi: 10.1073/pnas.1914017117. PMID: 31848247 PMC6955326

[271] Holth JK Fritschi SK Wang C Pedersen NP Cirrito JR Mahan TE . The sleep-wake cycle regulates brain interstitial fluid tau in mice and CSF tau in humans. Science. (2019) 363:880–4. doi: 10.1126/science.aav2546. PMID: 30679382 PMC6410369

[272] Cao X Xu H Feng W Su D Xiao M . Deletion of aquaporin-4 aggravates brain pathology after blocking of the meningeal lymphatic drainage. Brain Res Bull. (2018) 143:83–96. doi: 10.1016/j.brainresbull.2018.10.007. PMID: 30347264

[273] Hablitz LM Plá V Giannetto M Vinitsky HS Stæger FF Metcalfe T . Circadian control of brain glymphatic and lymphatic fluid flow. Nat Commun. (2020) 11:4411. doi: 10.1038/s41467-020-18115-2. PMID: 32879313 PMC7468152

[274] Lananna BV Nadarajah CJ Izumo M Cedeño MR Xiong DD Dimitry J . Cell-autonomous regulation of astrocyte activation by the circadian clock protein BMAL1. Cell Rep. (2018) 25:1–9.e5. doi: 10.1016/j.celrep.2018.09.015. PMID: 30282019 PMC6221830

[275] Dreha-Kulaczewski S Joseph AA Merboldt KD Ludwig HC Gärtner J Frahm J . Inspiration is the major regulator of human CSF flow. J Neurosci. (2015) 35:2485–91. doi: 10.1523/JNEUROSCI.3246-14.2015. PMID: 25673843 PMC6605608

[276] Liu X Hao J Yao E Cao J Zheng X Yao D . Polyunsaturated fatty acid supplement alleviates depression-incident cognitive dysfunction by protecting the cerebrovascular and glymphatic systems. Brain Behav Immun. (2020) 89:357–70. doi: 10.1016/j.bbi.2020.07.022. PMID: 32717402

[277] Wen J Satyanarayanan SK Li A Yan L Zhao Z Yuan Q . Unraveling the impact of omega-3 polyunsaturated fatty acids on blood-brain barrier (BBB) integrity and glymphatic function. Brain Behav Immun. (2024) 115:335–55. doi: 10.1016/j.bbi.2023.10.018. PMID: 37914102

[278] Gao H Findeis EL Culmone L Powell B Landschoot-Ward J Zacharek A . Early therapeutic effects of an Angiopoietin-1 mimetic peptide in middle-aged rats with vascular dementia. Front Aging Neurosci. (2023) 15:1180913. doi: 10.3389/fnagi.2023.1180913. PMID: 37304071 PMC10248134

[279] Wang FX Xu CL Su C Li J Lin JY . β-Hydroxybutyrate attenuates painful diabetic neuropathy via restoration of the aquaporin-4 polarity in the spinal glymphatic system. Front Neurosci. (2022) 16:926128. doi: 10.3389/fnins.2022.926128. PMID: 35898407 PMC9309893

[280] Chen C Zhu B Luo W Cao A Zhou W Weng Y . Trifluoperazine improves postoperative cognition by influencing astrocyte endfoot morphology and aquaporin-4 polarity. Mol Neurobiol. (2025) 62:12574–87. doi: 10.1007/s12035-025-05072-4. PMID: 40425909

[281] Jain S Malinowski M Chopra P Varshney V Deer TR . Intrathecal drug delivery for pain management: recent advances and future developments. Expert Opin Drug Delivery. (2019) 16:815–22. doi: 10.1080/17425247.2019.1642870. PMID: 31305165

[282] Aryal M Azadian MM Hart AR Macedo N Zhou Q Rosenthal EL . Noninvasive ultrasonic induction of cerebrospinal fluid flow enhances intrathecal drug delivery. J Ctrl Release. (2022) 349:434–42. doi: 10.1016/j.jconrel.2022.06.067. PMID: 35798095

[283] Wu C Lin T Ding Q Zhang N Ou ZT Cai GY . Continuous theta-burst stimulation promotes paravascular CSF-interstitial fluid exchange through regulation of aquaporin-4 polarization in APP/PS1 mice. Mediators Inflammation. (2022) 2022:2140524. doi: 10.1155/2022/2140524. PMID: 36032783 PMC9417777

[284] Yang CC Huang KY Hsu JL Hu CJ Lu YH Kuan YC . Effects of intermittent theta-burst stimulation on cognition and glymphatic system activity in mild cognitive impairment and very mild Alzheimer’s disease: a randomized controlled trial. J Neuroeng Rehabil. (2025) 22:195. doi: 10.1186/s12984-025-01738-1. PMID: 41013466 PMC12465570

[285] Lin GQ He XF Liu B Wei CY Tao R Yang P . Continuous theta burst stimulation ameliorates cognitive deficits in microinfarcts mice via inhibiting glial activation and promoting paravascular CSF-ISF exchange. Neuroscience. (2024) 561:20–9. doi: 10.1016/j.neuroscience.2024.09.046. PMID: 39366451

[286] Dai P Yu HX Wang ZX Liu SH Liu CB Xu GQ . Effect of continuous theta burst stimulation on the glymphatic system, brain network and cognitive function in patients with cerebral small vessel disease. Front Hum Neurosci. (2024) 18:1509483. doi: 10.3389/fnhum.2024.1509483. PMID: 39906273 PMC11790558

[287] Blanco-Duque C Chan D Kahn MC Murdock MH Tsai LH . Audiovisual gamma stimulation for the treatment of neurodegeneration. J Intern Med. (2024) 295:146–70. doi: 10.1111/joim.13755. PMID: 38115692 PMC10842797

[288] Murdock MH Yang CY Sun N Pao PC Blanco-Duque C Kahn MC . Multisensory gamma stimulation promotes glymphatic clearance of amyloid. Nature. (2024) 627:149–56. doi: 10.1038/s41586-024-07132-6. PMID: 38418876 PMC10917684

[289] Zhang S Zhou H Chen X Zhu S Chen D Luo D . Microneedle delivery platform integrated with codelivery nanoliposomes for effective and safe androgenetic alopecia treatment. ACS Appl Mater Iface. (2024) 16:15701–17. doi: 10.1021/acsami.3c16608. PMID: 38507687

[290] Zhao P Le Z Liu L Chen Y . Therapeutic delivery to the brain via the lymphatic vasculature. Nano Lett. (2020) 20:5415–20. doi: 10.1021/acs.nanolett.0c01806. PMID: 32510957

[291] Tong S Xie L Xie X Xu J You Y Sun Y . Nano-plumber reshapes glymphatic-lymphatic system to sustain microenvironment homeostasis and improve long-term prognosis after traumatic brain injury. Adv Sci Weinh. (2023) 10:e2304284. doi: 10.1002/advs.202304284. PMID: 37867233 PMC10700187

[292] Tavares GA Louveau A . Meningeal lymphatics: an immune gateway for the central nervous system. Cells. (2021) 10:3385. doi: 10.3390/cells10123385. PMID: 34943894 PMC8699870

[293] Kim MW Kipnis J . Glymphatics and meningeal lymphatics unlock the brain-immune code. Immunity. (2025) 58:1040–51. doi: 10.1016/j.immuni.2025.03.006. PMID: 40324376 PMC12866980

[294] Mao L Wang L Huang Z Switzer JA Hess DC Zhang Q . Perioperative neurocognitive disorders: advances in molecular mechanisms and bioactive molecules. Ageing Res Rev. (2025) 112:102885. doi: 10.1016/j.arr.2025.102885. PMID: 40914484 PMC12825322

[295] Ballweg T White M Parker M Casey C Bo A Farahbakhsh Z . Association between plasma tau and postoperative delirium incidence and severity: a prospective observational study. Br J Anaesth. (2021) 126:458–66. doi: 10.1016/j.bja.2020.08.061. PMID: 33228978 PMC8014913

[296] Cui H Wang W Zheng X Xia D Liu H Qin C . Decreased AQP4 expression aggravates α-synuclein pathology in Parkinson's disease mice, possibly via impaired glymphatic clearance. J Mol Neurosci. (2021) 71:2500–13. doi: 10.1007/s12031-021-01836-4. PMID: 33772424

[297] Bostancıklıoğlu M . SARS-CoV2 entry and spread in the lymphatic drainage system of the brain. Brain Behav Immun. (2020) 87:122–3. doi: 10.1016/j.bbi.2020.04.080. PMID: 32360606 PMC7189839

[298] Ren X Liu S Lian C Li H Li K Li L . Dysfunction of the glymphatic system as a potential mechanism of perioperative neurocognitive disorders. Front Aging Neurosci. (2021) 13:659457. doi: 10.3389/fnagi.2021.659457. PMID: 34163349 PMC8215113

[299] Jia L Chen Y Li H Zhao K Ge S Wang C . The glymphatic system in neurodegenerative diseases and brain tumors: mechanistic insights, biomarker advances, and therapeutic opportunities. Acta Neuropathol Commun. (2025) 14:19. doi: 10.1186/s40478-025-02203-9. PMID: 41390476 PMC12821863

[300] Oxford Centre for Evidence-Based Medicine . The 2011 Oxford CEBM levels of evidence (introductory document) (2011). Available online at: https://www.cebm.ox.ac.uk/resources/levels-of-evidence/ocebm-levels-of-evidence (Accessed March 30, 2026).

[301] Dilmen OK Meco BC Evered LA Radtke FM . Postoperative neurocognitive disorders: a clinical guide. J Clin Anesth. (2024) 92:111320. doi: 10.1016/j.jclinane.2023.111320. PMID: 37944401

[302] Liu X Xie Y Wan X Wu J Fan Z Yang L . Protective effects of aquaporin-4 deficiency on longer-term neurological outcomes in a mouse model. Neurochem Res. (2021) 46:1380–9. doi: 10.1007/s11064-021-03272-7. PMID: 33651262

[303] Yu H Kang H Fan J Cao G Liu B . Influence of dexmedetomidine on postoperative cognitive dysfunction in the elderly: a meta‐analysis of randomized controlled trials. Brain Behav. (2022) 12:e2665. doi: 10.1002/brb3.2665. PMID: 35810480 PMC9392542

[304] Zhang X Leng Y Yuan X Yang Y Zhou C Liu H . Efficacy of perioperative dexmedetomidine in postoperative pain and neurocognitive functions in orthopedic surgery: a systematic review and meta-analysis with trial sequential analysis of randomized controlled trials. Int J Surg. (2025) 111:3525–42. doi: 10.1097/JS9.0000000000002315. PMID: 40042401 PMC12165510

[305] Duan X Coburn M Rossaint R Sanders RD Waesberghe JV Kowark A . Efficacy of perioperative dexmedetomidine on postoperative delirium: systematic review and meta-analysis with trial sequential analysis of randomised controlled trials. Br J Anaesth. (2018) 121:384–97. doi: 10.1016/j.bja.2018.04.046. PMID: 30032877

[306] Huang X Quan Z Zhan C Sandeep B Bu J . Perioperative neurocognitive disorder in colorectal cancer surgery: a systematic review of incidence, mechanisms, and interventions. Front Surg. (2025) 12:1698597. doi: 10.3389/fsurg.2025.1698597. PMID: 41340990 PMC12669154

[307] Liu Y Dong Y Wang X Huang Y Wu F Xia F . Effects of lavender essential oil inhalation aromatherapy on postoperative sleep quality in patients with intracranial tumors: a randomized controlled trial. Front Pharmacol. (2025) 16:1584998. doi: 10.3389/fphar.2025.1584998. PMID: 40832605 PMC12358394

[308] Han S Cai Z Cao L Li J Huang L . Effects of Chinese traditional five-element music intervention on postoperative delirium and sleep quality in elderly patients after non-cardiac surgery: a randomized controlled trial. Perioper Med. (2024) 13:47. doi: 10.1186/s13741-024-00408-5. PMID: 38807220 PMC11134639

[309] Shin HW Kwak JS Choi YJ Kim JW You HS Shin HJ . Efficacy and safety of perioperative melatonin for postoperative delirium in patients undergoing surgery: a systematic review and meta-analysis. J Int Med Res. (2024) 52:3000605241239854. doi: 10.1177/03000605241239854. PMID: 38735057 PMC11089947

[310] Tang X Li J Yang B Lei C Dong H . Efficacy of sleep interventions on postoperative delirium: a systematic review and meta-analysis of randomized controlled trials. Anesthesiol Perioper Sci. (2023) 1:29. doi: 10.1007/s44254-023-00027-1. PMID: 41933263

[311] Ludwig R Rippee M D’Silva L Radel J Eakman AM Morris J . The impact of cognitive behavioral therapy for insomnia on neurofilament light and phosphorylated tau in individuals with a concussion. Arch Clin Neuropsychol. (2025) 40:437–44. doi: 10.1093/arclin/acae096. PMID: 39504933 PMC12034518

[312] Guo Z Tang X Zhong S Chen G Chen P Chen C . Vortioxetine improves brain glymphatic system function, functional connectivity, and cognitive functions in major depressive disorder. Depress Anxiety. (2025) 2025:1990117. doi: 10.1155/da/1990117. PMID: 40917304 PMC12413946

[313] Zhang X Sun Z Wu D Shi X Song C Guan X . Effects of cerebellar intermittent theta-burst stimulation on patients with Alzheimer’s disease: a randomized controlled trial. J Alzheimers Dis. (2025) 107:1187–99. doi: 10.1177/13872877251366656. PMID: 40801847

[314] Dong Y Liu J Wang T Zhang Z Hu X Zhu C . Repetitive transcranial magnetic stimulation promotes the recovery of upper limb motor dysfunction in ischemic stroke patients: a DTI-based glymphatic system imaging prospective study. PeerJ. (2026) 14:e20709. doi: 10.7717/peerj.20709. PMID: 41660074 PMC12880090

[315] Wang J Tian Y Qin C Meng L Feng R Xu S . Impaired glymphatic drainage underlying obstructive sleep apnea is associated with cognitive dysfunction. J Neurol. (2023) 270:2204–16. doi: 10.1007/s00415-022-11530-z. PMID: 36662283 PMC10025229

